# Single-cell mRNA isoform diversity in the mouse brain

**DOI:** 10.1186/s12864-017-3528-6

**Published:** 2017-02-03

**Authors:** Kasper Karlsson, Sten Linnarsson

**Affiliations:** 1 Departments of Medicine and Genetics, Stanford University, 94305 Stanford, CA USA; 2 Laboratory for Molecular Neurobiology, Department of Medical Biochemistry and Biophysics, Karolinska Institutet, Scheeles väg 1, SE-171 77 Stockholm, Sweden

**Keywords:** Alternative isoform usage, Single-cell RNA sequencing, STRT, PacBio, Long read sequencing, UMI, Oligodendrocytes

## Abstract

**Background:**

Alternative mRNA isoform usage is an important source of protein diversity in mammalian cells. This phenomenon has been extensively studied in bulk tissues, however, it remains unclear how this diversity is reflected in single cells.

**Results:**

Here we use long-read sequencing technology combined with unique molecular identifiers (UMIs) to reveal patterns of alternative full-length isoform expression in single cells from the mouse brain. We found a surprising amount of isoform diversity, even after applying a conservative definition of what constitutes an isoform. Genes tend to have one or a few isoforms highly expressed and a larger number of isoforms expressed at a low level. However, for many genes, nearly every sequenced mRNA molecule was unique, and many events affected coding regions suggesting previously unknown protein diversity in single cells. Exon junctions in coding regions were less prone to splicing errors than those in non-coding regions, indicating purifying selection on splice donor and acceptor efficiency.

**Conclusions:**

Our findings indicate that mRNA isoform diversity is an important source of biological variability also in single cells.

**Electronic supplementary material:**

The online version of this article (doi:10.1186/s12864-017-3528-6) contains supplementary material, which is available to authorized users.

## Background

Alternative mRNA isoform usage is prevalent in mammalian genomes, and allows the creation of a highly diverse set of proteins from a relatively small number of genes. The phenomenon was first described around 30 years ago [1, 2], and was initially thought to be rare. With the introduction of mRNA sequencing technology, today we know that more than 90% of all multi-exon genes in humans are alternatively spliced [3, 4].

Alternative isoform usage has been studied at the 5′ end [5]; for exon splicing [3] and at the 3′ end [6]. Furthermore, exon splicing can be divided into four categories: alternative 5′ splice-site choice, alternative 3′ splice-site choice, cassette-exon inclusion and intron retention [7]. Currently the most common approach to study alternative isoform usage is by second generation sequencing, but some studies have also been performed using imaging [8]. Most studies have used bulk tissue, which contains a mixture of cell types, but a growing number of publications study isoform usage at the single cell level, e.g., 3′ polyadenylation [9] and exon-cassette inclusion [10], but not yet full-length isoforms. There has also been some investigation into the potential long-range correlation of multiple exon-cassette inclusion events [11, 12] as well as correlation between transcription start site (TSS) and transcription termination site (TTS) [13].

Most studies have found a considerable diversity of isoforms. A recent paper [14] from the Encode consortium combining RNA-seq, CAGE and paired end tags found that cell lines tend to express genes as multiple isoforms simultaneously and that the number of isoforms per gene grew with increased number of annotated isoforms. Similarly, a study using long-read sequencing showed that the majority of genes are alternatively spliced [15]. However, since these studies were based on bulk material, it remains unknown whether multiple isoforms are also present in each individual cell.

Alternative isoform usage has also been shown to have functional importance [7] and to be dysregulated in disease, e.g., in cancer [16] and Alzheimer’s disease [17]. Extreme examples of isoform diversity include the Drosophila Dscam1 gene, which produces more than 38,000 isoforms [12] and is required for neurite self-avoidance in the wiring of the Drosophila nervous system. In mammals, a similar function may be served by the clustered protocadherins, which generate isoform diversity through the use of large numbers of alternative promoters and first exons [18].

Most previous studies of isoform diversity have relied on short-read sequencing (which cannot define full-length isoforms) of bulk samples (which cannot determine isoform usage in individual cells). However, recent progress in DNA sequencing technology now allows full-length end-to-end sequencing of cDNA. Similar progress in sample preparation now permits the generation of high-quality full-length cDNA from single cells. Here we take advantage of the PacBio long read sequencing technology and the precision allowed by using unique molecular identifiers (UMI) [19] to get a comprehensive understanding of alternative isoform usage at the single cell level. PacBio long read sequencing provides exceptionally long reads, in our case up to 5000 bases, but comes at a cost of lower throughput. PacBio sequencing allowed us to study all aspects of isoform usage (TSS, TTS, exon-cassette inclusion, intron retention and exon 5′ and 3′ position) in cDNA amplified from single primary cells. Although amplification can introduce both quantitative bias and artefactual mutations, we were able to control and manage these sources of error using unique molecular identifiers (UMIs). This technology was instrumental since it allowed both identification and counting of individual cDNA molecules [19], and correct sequencing errors [20, 21].

We find that a large fraction of all transcripts in single cells constitute distinct isoforms (in average 1.7 transcripts per conservative isoform). We also show that single-cell isoform diversity affects the protein coding repertoire: genes in single cells commonly have more than three coding isoforms and in extreme cases more than 20 coding isoforms.

Most of the diversity is created at the 5′ and 3′ ends of the transcript, but a substantial amount of diversity is also created by alternative exon cassette inclusion as well as shifts in the location of exon 5′ and 3′ splice sites. Furthermore, we show that exon junctions in coding regions are less diverse than exon junctions in non-coding regions of transcripts, suggesting purifying selection against coding variants.

## Results and discussion

### Measuring isoform diversity in single cells

We selected six single cells for which cDNA was available from an earlier experiment [22]. Two cells were vascular and leptomeningeal cells (VLMCs), and four cells represented stages of oligodendrocyte maturation: Oligo1 (immature oligodendrocyte) and Oligo5 (mature myelinating oligodendrocyte). The cDNA was previously prepared using STRT/C1 [23], which resulted in full-length cDNA normally sequenced from the 5′ end, to indicate only the transcription start site. Here, we instead sequenced each cDNA sample using Pacific Biosciences Single Molecule Real Time (PacBio SMRT) technology [24], which generated long reads often comprising the entire length of each cDNA molecule. Known adapter sequences were trimmed off each end and their presence was used to confirm the full-length nature of each read. Two PacBio runs were performed, the second of which used an enrichment step for long molecules and an improved sample preparation method and yielded longer reads; reads from both experiments were pooled. We found that a large number of reads in the long data set consisted of concatemers of shorter molecules (33% of all molecules in the long data set contained three or more detectable cDNA ends, as shown by the presence of adapter sequences). This phenomenon was also present in the short data set, albeit much less frequently. Since samples were pooled after PCR amplification but before circularization, and since fragments were always found ligated end to end, we conclude that the concatemerization must have happened during the circularization reaction. We therefore split such reads into individual subreads using the adapter sequences. In order to ensure read length was not limiting, we removed all reads that did not include the polyadenylation tail as well as the first exon (Additional file 1: Figure S2A-B). Due to this, the relatively low read depth and the generally low transcript capture efficiency of single cell RNA sequencing protocols, the results below are lower-bound estimates of isoform diversity. A summary of the six sequenced cDNA libraries is given in Additional file 1: Figure S1, Additional file 2: Table S1 Reads per cell, Additional file 3: Table S2 Summary Conservative Isoforms, Additional file 4: All isoforms and Additional file 5: Isoforms and coding isoforms per gene.

We next analyzed the technical performance of our methods, to determine their quantitative accuracy. PacBio sequencing of single-cell RNA requires extensive amplification so there was a concern that the amplification would cause bias in the data. We used unique molecular identifiers (UMIs) to label individual cDNA molecules before PCR, and hence to identify and merge redundant PacBio reads originating from the same original cDNA molecule. Due to the low read depth from PacBio sequencing a large number of transcripts constituted singletons, i.e., UMIs observed only once (61% of the total). We opted to not remove such molecules since due to the low sequencing depth, they are likely to represent true isoforms. However, we made use of those cases where UMIs were sampled more than once to assess the technical reproducibility of our methods. In this way, we were able to correct for unequal amplification, as well as correct sequencing errors that otherwise would have resulted in spurious false-positive isoforms. Since each read with the same UMI came from the same molecule it was also possible to assess these technical artefacts by analyzing how much variability there was between reads with the same UMI.

We found very few errors at exon junctions (<1% of reads offset by 1 bp; Additional file 1: Figure S3A-C), however the variability at the 5′ and 3′ ends was higher, but mostly restricted to an offset of 1 bp (<15% of reads offset by 1 bp for ERCC reads and slightly higher for endogenous genes). The variability at the 5′ end was slightly lower than at the 3′ end, perhaps reflecting the presence of a polyguanine stretch at the 5′ end of these cDNAs (introduced during cDNA synthesis). Overall however, we found that we were confidently able to measure 5′ and 3′ ends of cDNAs, as well as exon-exon junctions with an accuracy of about ±1 bp. Note that the errors reported here are raw errors before UMI correction. We merged all reads with the same UMI by taking the consensus start and end position of each exon, thus reducing the error.

**Fig. 1:**
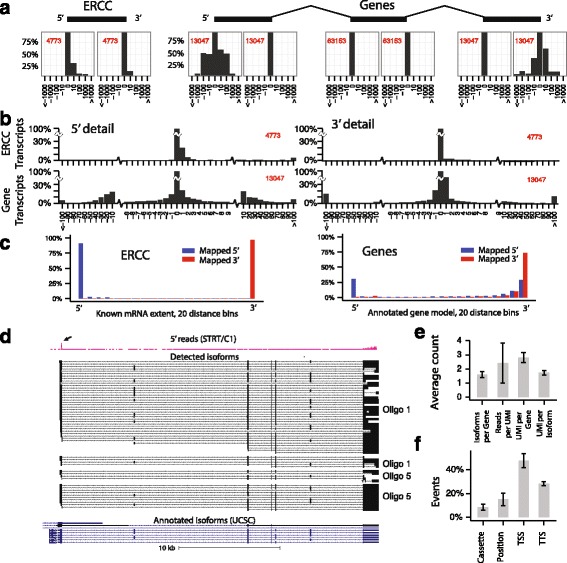
Full-length mRNA isoforms in single cells. **a** Offset from the median mapped position for ERCC transcripts (*left*) and endogenous genes (*right*). Histograms showing the distribution of offsets for 5′ and 3′ ends, and internal splice junctions, as percentage of the count for zero offset. Horizontal axis shows offset in base pairs (note that the axis has variable bin sizes). *Red*, the total number of events used to create each plot. **b** Magnification of the 5′ and 3′ end for both ERCC transcripts and transcripts mapping to genes. Note that the bin sizes are larger after ±10 bp. **c** Relative position of transcript ends for ERCC (*left*) and endogenous genes (*right*). **d** Isoforms observed for *Mbp*. Each *black track* is a separate isoform (not a separate UMI). *Pink track*, reads from Zeisel et al. [22], obtained using a 5′-specific RNA-seq method. The *arrow* points to a peak representing the transcript start site from this extended dataset. *Blue tracks*, UCSC gene models. **e** Average number of isoforms per gene, reads per UMI, UMI per gene and UMI per isoform. Error bars, standard deviation. **f** Distribution of isoform event types. *Error bars* show standard deviations

Another possible source of error is reverse transcription. When two identical mRNA molecules are reverse transcribed, it is possible that one of them does not result in a full-length cDNA, which could be mistaken for a true mRNA isoform. UMIs cannot correct for such errors, since UMIs are introduced during cDNA synthesis (and will thus label the two cDNA molecules with two distinct UMIs). To measure this source of technical error, we examined the ERCC (External RNA Controls Consortium) spike-in control RNA, which comprise 92 commercially available *in vitro* transcribed synthetic mRNAs, ranging from 255 to 2007 bp in length. ERCC transcripts have known start and end positions and can therefore be used as a benchmark for how frequently cDNAs include the proper 5′ and 3′ ends. We calculated the offset from the expected 5′ and 3′ positions for each ERCC transcript. As expected, it was greater than the sequencing error alone, with most reads falling within ±5 bp (Fig. 1a and b) both at the 5′ and the 3′ ends. The 3′ end was slightly more accurate than the 5′ end, probably reflecting premature termination of reverse transcription resulting in a shorter molecule with correct 3′ but truncated 5′ end. Ninety-two percent of all reads were aligned to within 5 bp of the 5′ end, and 98% within 5 bp of the 3′ end (Fig. 1b and c).

In previous work using PacBio sequencing [15], a marked drop in read length was noticed for ERCC molecules longer than 1.5 kb, where a median of 377 base pairs was missing. However, in our present dataset the median number of missed nucleotides for all ERCC transcripts, for both 5′ and 3′ positions, was zero, and the number of transcripts that deviated from the correct ERCC starting position increased only slightly with increased transcript length, as shown in Additional file 1: Figure S4. In conclusion, technical sources of error introduced an uncertainty of around ±1 bp at each exon boundary and less than ±5 bp transcript 5′ and 3′ ends. Conservatively, we therefore considered all variability within these boundaries as technical artifacts, which were omitted from all analyses below, and we restricted our analysis to transcripts that both covered an annotated first exon and contained a poly-A tail to ensure full length isoforms are studied.

### Isoform structure in single cells

First, we examined the transcription start and termination sites. As expected, ERCC control RNAs were nearly all full-length and both 5′ and 3′ ends mapped to the extremes of each transcript (Fig. 1c). In contrast, for endogenous genes only around 30% of 5′ ends of transcripts were located near the annotated 5′ end, with a large number of truncated transcripts aligned to the 3′ UTR. This was in agreement with our previous finding that endogenous genes tend to be truncated at their 5′ ends, probably partly representing ongoing mRNA degradation [25], partly the presence of unannotated alternative transcription start sites, and partly due to strand invasion during reverse transcription, which has been shown to contribute with template switching artifacts [26].

Similarly, only around 70% of 3′ ends were located at the annotated 3′ end of genes, with the remainder distributed mostly in the 3′ UTR but away from the annotated transcription termination site. Thus, most truncated 3′ ends could be attributed to alternative polyadenylation sites in the 3′ UTR (or to degradation from the 3′ end). However, interestingly almost 5% of all transcripts ended close to the annotated 5′ end of the gene (within the first 15% of the total gene length, Fig. 1c), thus likely representing short prematurely terminated transcripts.

To illustrate the extent of isoform diversity, and the structure of common isoforms, we visualized isoforms of the *Mbp* gene (Myelin basic protein; Fig. 1d). Because this gene is highly expressed in oligodendrocytes, it clearly shows a number of commonly occurring features. It showed multiple different TSSs in the first exon, as well as probably degradation from the 5′ end (although it cannot be excluded that some of those events are alternative TSSs). The heterogeneity in the 3′ UTR was even greater, in the form of 3′ truncations as well as exclusion of internal segments of the 3′ UTR. As seen in Additional file 1: Figure S5, this phenomenon appears also in other highly expressed genes like *Plp1* and *Cnp*. There were a number of different exon cassette inclusion events. Interestingly, *Mbp* showed evidence of exon connectivity [11], where exons 4 and 5 were almost always either both included or both excluded (*P* < 0.001 by Fisher’s exact test, two-sided). This was in contrast to exons 2 and 3, which were independently excluded or included (*P* = 0.31). Overall, nearly every *Mbp* transcript was different (for example 61 transcripts distributed over 33 isoforms for Oligo 1.1, as seen in Additional file 5), and this diversity existed within individual oligodendrocyte cells.

We validated that the identified exon isoforms weren’t an artefact of the sequencing process by Sanger sequencing a total of 26 isoforms (represented by more than UMI) from 5 genes. To see if the isoform results were reproducible in a cell that hadn’t been sequenced an “independent” cell was added to the validation experiments. Additionally, to verify that the isoforms identified weren’t an artefact of the amplification process, an amplification-free library was created of bulk material from oligodendrocyte rich areas of the brain. The amplification-free sequencing could verify full-length isoforms, whereas Sanger sequencing verified inclusion/exclusion of specific exon. The results are shown in Additional file 1: Figure S5A-J. As an example, Additional file 1: Figure S5E shows the *Mbp* gene, the primer pairs (PP) used in the PCR, the length of the PCR product for two cells and the mapping of the Sanger sequenced PCR products. A number of conclusions can be drawn from these validation experiments. First they confirm the results from PacBio sequencing: Exon cassette 2 can either be included or excluded (PP 1, 2 and 6), exon cassettes 3, 4 and 5 seem to be included or excluded in combination (PP 2), exon cassette 6 can either be included or excluded (PP 4), and extensive heterogeneity is seen in the 3′ UTR, where some isoforms excludes a large part of exon 7 (PP 5). Interestingly, the two cells used for validation gave very similar results, the most obvious difference being for PP 5 where oligo 1.1 has two bands and oligo 1.2 has three. One of the shorter products (PP5-C in Additional file 1: Figure S5E (A and D)) for oligo 1.2 was verified by Sanger sequencing, as well as the shorter product for oligo 1.1, and those two products were different. Unsurprisingly this heterogeneity was in the UTR region. Thus, the differences in isoforms observed by PacBio sequencing between cells oligo 1.1 and oligo 1.2 are probably partly due to incomplete sequencing of the latter cell, although some isoforms were clearly cell-specific. This also shows that the number of isoforms identified in this study is a lower-bound estimate, especially for cells with fewer sequencing reads.

Unfortunately, the amplification-free library for PacBio sequencing had markedly fewer accepted Zero-mode waveguides (ZMW) compared to the libraries from single cells (in average 7.500 compared to 22.500 for the long read long read and 28.500 for the short read library), using standard quality filtering. The library was sequenced two times and the loading concentration was increased the second time, but that didn’t increase the number of accepted ZMWs. We therefore lowered the calling stringency from one pass (reads with adapter sequence at both ends) and a quality score of 90, to zero pass and a quality score of 75 for the amplification-free library, since large structures like exons still would be identified even with lower quality sequences. This increased the number of accepted ZMWs to in total 45.100. Still some highly expressed genes in the single-cell data set had no or very few transcripts in the amplification-free data set, like *Mobp*, were not a single isoform could be confirmed.

Generally, exon structures with many transcripts could be verified with both Sanger sequencing and amplification-free Pacbio sequencing. Sanger sequencing could verify more isoforms than amplification-free sequencing probably due to the low read depth of the amplification-free library, in combination with that the library was made of bulk material, including many non-oligodendrocyte cells, and was prepared from the brain stem and striatum (due to high oligodendrocyte content) compared with the single cells that came from hippocampus and the cortex. Of 26 exon isoforms identified in the 5 genes that were Sanger sequenced (single transcript exon isoforms not counted), 8 could be identifies by both methods, 9 more could be identified by Sanger sequencing and 9 couldn’t be verified by any method. Interestingly the existence of 3′UTR introns for some genes could be verified by both Sanger sequencing and amplification-free Pacbio sequencing.

Not all genes were as highly expressed as *Mbp*. Average number of transcripts per gene were just three, and average number of UMI per isoform two (Fig. 1e). To quantify the sources of isoform diversity in single cells, we counted cases of alternative “TSS” (5′ end of the cDNA), “TTS” (3′ end of the cDNA), “position” (differences in the start or end position of an exon) and “cassette” (exon cassette inclusion/exclusion). We occasionally observed intron retention, but they were very rare events and were therefore excluded from further analysis. As shown in Additional file 1: Figure S3, the variability at exon 5′ and 3′ borders was similar, and we therefore combined these into a single “position” category. Alternative TSS and TTS variation represented more than 70% of all isoform-generating events (Fig. 1f). In contrast, exon cassette exclusion and exon position events affected only about 10–20% of all events. Like *Mbp*, many genes were affected by all sources of isoform diversity, which led to a very large number of distinct isoforms in single cells. There was a high variation in number of events per cell, which reflected differences in the total number of mRNA molecules. However, the ratio between the events was stable among the cells.

**Fig. 2:**
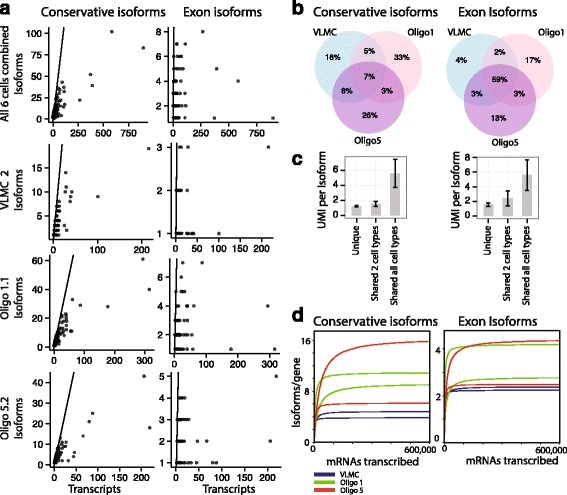
Heterogeneity of isoforms among single cells. **a** Number of distinct isoforms as a function of the number of observed transcripts, for pooled single cells (*top*) and individual single cells. Each dot is a gene. *Left column*, conservative isoforms. *Right column*, exon cassette isoforms only. *Black line* indicates where the number of isoforms equals the number of transcripts. **b** Venn diagrams showing the number of shared isoforms between the three different cell-types. *Left*, conservative isoforms. *Right*, exon cassette isoforms only. **c** Histogram showing the number of transcripts per isoform for shared and unique isoforms. **d** Extrapolation of the number of conservative isoforms per cell (*left*) and the number of exon cassette isoforms per cell (*right*)

Thus it is clear that the combination of several sources of diversity leads to a great heterogeneity of mRNA isoforms, even in single cells. Intriguingly, we found that for many genes nearly every single transcript represented a distinct isoform (e.g., *Mbp*). As gene expression levels increased, the number of isoforms increased almost as rapidly. This was true for pooled data as well as within individual single cells (Fig. 2a), however it was not true when considering exon cassette isoforms only, where no such trend could be discerned.

Few isoforms were shared between cells as shown in Fig. 2b. Only 23% of all detected isoforms were shared between any two cell-types, and only 7% of all detected isoforms were shared between all cell-types (considering only isoforms belonging to genes represented by at least one transcript in all cell types). These are lower-bound estimates, because of the limited depth of sequencing, and (as noted above) validation by Sanger sequencing showed a greater proportion of shared splicing events.

For exon cassette isoforms, almost 60% of all detected isoforms were shared. The expression level was generally higher for shared isoforms (Fig. 2c). However lowly expressed isoforms have a higher probability to be missed due to the low mRNA capture rate, so it is possible that lowly expressed isoforms are shared among the different cell types too. The major isoform constituted in average around 50% of total gene expression (considering only genes with more than 10 transcripts, Additional file 1: Figure S6A), suggesting that some isoforms were preferred. Neither the number of annotated gene exons nor the overall gene expression had a major impact on the percentage of major isoform expression (Additional file 1: Figure S6 B-C).

In order to estimate the true number of isoforms in each single cell, we made use of a recently published Bayesian method to accurately extrapolate the complexity of DNA libraries (Preseq, [27]). We found that when a cell over time has transcribed 600,000 molecules of mRNA, it will have generated between 5 and 15 conservative isoforms per gene, and between 2 and 4 exon cassette isoforms (Fig. 2d). Both VLMC cells showed a low estimated number of isoforms per gene, which is reasonable considering that VLMCs are small and express a smaller total number of mRNA molecules.

**Fig. 3:**
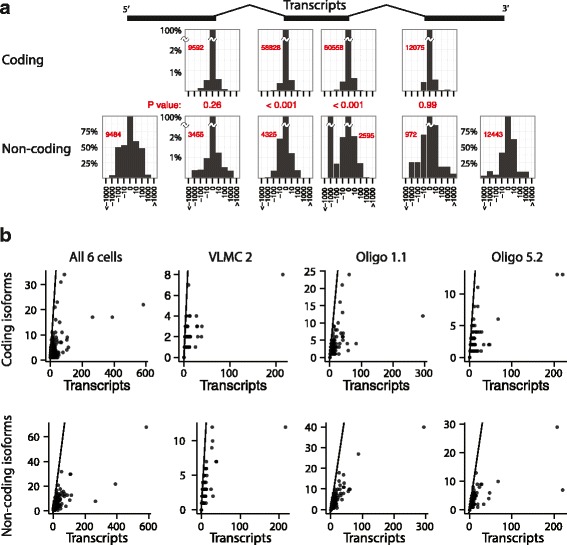
Isoforms in coding and non-coding regions. **a** Histograms showing the distribution of offsets for 5′ and 3′ ends, and internal splice junctions, as percentage of the count for zero offset. Horizontal axis shows offset in base pairs (note that the axis has variable bin sizes). *Red*, the total number of events used to create each plot. Note, this is the same figure as Fig. 1a but it shows coding and noncoding exons separately. **b** Number of conservative coding and non-coding isoforms for each gene, as a function of the observed number of transcripts

The observation of a great diversity of isoforms in single cells naturally leads to the question of how this may affect the repertoire of proteins expressed. Isoform diversity was not limited to non-coding regions, as can be seen in Fig. 3b, which shows isoform diversity considering only coding isoforms (Methods). Thus, even in single cells, each gene can be expected to give rise to multiple distinct protein isoforms, greatly expanding the coding repertoire.

We hypothesized that isoforms that would affect protein-coding sequence would be more tightly regulated, leading to a reduced diversity at these sites. To examine this, we repeated the analysis leading up to Fig. 1a, splitting the dataset into coding and non-coding events (Fig. 3a; see Methods). There was a clear difference between coding and noncoding splicing events (Fig. 3a and Additional file 1: Figure S7). The observed variation in coding exon junctions was limited to ±1 bp, in the same range as the technical variation due to amplification or sequencing errors (Additional file 1: Figure S3 and S7). In contrast, at non-coding exon junctions the variation was larger, extending well outside the ±1 bp region, and sometimes as far as hundreds of base pairs. The difference between coding and non-coding junctions was statistically significant (*P* < 0.001 Wilcoxon signed-rank test, two-sided) both at the start and end of internal exons. This suggests that coding exon splicing has evolved to be under stricter control than non-coding exon splicing, likely to prevent the generation of anomalous protein products.

Despite the stricter control of coding variants at each splice junction, since genes contain multiple exons, we found that a large number of coding isoforms were present in single cells (Fig. 3b). Although most genes had fewer than five isoforms, many had more and even under conservative estimates some genes showed up to 25 distinct coding isoforms. These results point to an underappreciated richness of alternative protein forms being simultaneously present in individual cells.

## Conclusions

We have studied single-cell oligodendrocyte transcriptomes using long-read PacBio sequencing technology at unprecedented accuracy due to the use of UMIs. The most striking finding was the large number of separate isoforms present in single cells. For example, VLMC-2 (a cell that was sequenced to reasonable saturation) contained about 2000 unique conservative transcripts mapped to around 700 genes and 1000 distinct isoforms (as seen in Additional file 3: Table S2). Generally, the higher the expression level of a gene, the more isoforms were observed, and for many genes the number of isoforms grew almost linearly with expression level (Fig. 2a).

As a consequence of this diversity, there was little sharing of isoforms between cells of different type (Fig. 2b). However, the isoforms that were shared between cell types were more highly expressed (Fig. 2c) and the major isoform for a gene constituted around 50% of all expression (Additional file 1: Figure S7A), indicating a preference for certain isoforms.

Non-coding isoforms (of coding genes) are less likely to influence phenotype than coding isoforms, even if noncoding isoforms may be subject to differential processing and degradation. Intriguingly, coding exon junctions were less variable than non-coding junctions, demonstrating a purifying selection against coding variants that must have refined splice donor/acceptor signals at coding sites.

In conclusion, we have shown that single cells harbor a great diversity of mRNA isoforms, revealing a source of stochasticity between putatively identical cells. Such heterogeneity could contribute to our understanding of phenotypic heterogeneity such as drug resistance.

## Methods

### PacBio sequencing

Stored cDNA from an earlier single cell experiment [22] was used for PacBio sequencing. The cDNA had been produced with the STRT method adapted to the Fluidigm C1 instrument for single cell RNA sequencing. The cDNA was first diluted 1:10 or 1:20 and then additionally amplified 12–15 rounds using primers with a 16 bp barcode, leading to a total amplification of 33–36 rounds. The amplification was done with Advantage polymerase (Clontech) in the following buffer: 2 μl cDNA, 2,5 μl 10× Advantage buffer, 1 μl 10 mM dNTP, 1,5 μl 10 μM PacBio index primers (idx 1-6), 1 μl Advantage polymerase, 17 μl water. The PCR was cycled with 1 min 95 °C, then 12 to 15 cycles of 95 °C 30 s, 64 °C 30 s and 68 °C 7 min, then 1 round of 72 °C for 7 min. The samples were purified with Ampure beads 1× and then run on the Bioanalyzer for quality control. The samples were pooled and gel purified (Qiagen gel extraction kit), molecules shorter than 500 bp were discarded. Unfortunately, not all cells amplified equally well and for two cells less material was used in the final library. A total amount of 1.5 μg DNA was sent to the PacBio Sequencing Services at the University of Washington to be sequenced at an RSII instrument. A total of 7 SMRT cells were sequenced for single cells. The first 5 SMRT cells were done with dilution loading and P4/C2 chemistry. After the first 5 SMRT cells another round of Ampure purification 0.45× was done to enrich for longer fragments and the loading was done with MagBeads and P6/C4 chemistry. There was a marked difference in terms of read length between the first 5 SMRT cells and the last 2 as can be seen in Additional file 1: Figure S1. Additionally, 2 SMRT cells were sequenced using bulk material from mouse brain stem and striatum. This library was prepared without amplification. RNA was extracted with Trizol (according to manufacturer’s intructions) and the library was prepared with a scaled-up version of STRT for single cells. Superscript II was used for mRNA capture and reverse transcription as follows: 1 ul 100 μM oligo C1-P1-T31 was mixed with 3 μl DNA (around 5 μg total RNA) and 1 μl dNTP (10 mM). The sample was incubated at 65 °C for 5 min and put on ice. Then 4 μl 5× First Strand Buffer and 2 μl 0.1 M DTT was added, and the sample was incubated at 42 °C for 3 min. The 1 μl of 100 μM C1-P1-RNA-TSO and 1 μl Superscript II was added and the sample was incubated at 42 °C for 90 min and then 70 °C for 10 min. After reverse transcription 1 μl RNAse cocktail (Ambion) was added and the sample was incubated at 37 °C for 20 min. Two cycles of PCR were done using the Advantage PCR polymerase (which company) using the following buffer: 20 μl cDNA, 5 μl 10× Advantage buffer, 1 μl 10 mM dNTP, 2 μl 10 μM PacBio index primers (idx 2 for Striatum and idx3 for Brain stem), 1 μl 50× Advantage polymerase and 21 μl water. The PCR was cycled with 1 min 95 °C, then 2 cycles of 95 °C 30 s, 58 °C 4 min and 68 °C 7 min, then 1 round of 72 °C for 10 min. Samples were pooled and cleaned using Ampure beads 0.6×, and the quality of the pooled sample was checked using the Bioanalyzer.

## Analysis

### Alignment

Quality of PacBio reads was examined with FastQC [28].

Only circular consensus sequences were used in the analysis. By careful analysis of the fastq file it was discovered that a large number of reads were concatamers, as shown in Additional file 1: Figure S9-10. 3% of the short reads and 33% of the long reads contained matches to three or more adaptor sequences, allowing for 2 mismatches. Since concatamers contained molecules from different samples we concluded that the concatamerization must have happened in the circularization step, not during PCR. This feature of the PacBio reads demanded special handling. Each pre-circularization molecule should have the following sequences or their reverse complement depending on which strand was read: Specific PacBio barcode, Illumina adaptor (AATGATACGGCGACCACCGAT), UMI, GGG, template molecule, poly-A, Illumina reverse complement and the same PacBio barcode reverse complement. This was called “case 1”. If the other strand was read the order would be: Specific PacBio barcode, Illumina adaptor, polyT, template molecule, CCC, UMI reverse complement, Illumina adaptor reverse complement and PacBio barcode reverse complement. This was called “case 2”.

A read was considered valid if the specific PacBio barcode and its reverse complement were identified and no other barcode was found in between. Further in case 1 the PacBio barcode must be in proximity to the Illumina adapter and the reverse complement of the PacBio barcode must be in proximity to the reverse complement of the Illumina adapter and a string of 10 A. Here the UMI was identified as the 6 bases after the end of the Illumina adapter. In case 2 the PacBio barcode must be in proximity to the Illumina adapter and a string of 10 T, and the PacBio barcode reverse complement must be in proximity to the reverse complement of the Illumina adapter. Here the UMI was defined as the reverse complement of the 6 bases just before the start of the reverse complement of the Illumina adapter. Proximity was here defined as ±10 bp. Valid reads that contained both a poly-A stretch and a poly-T stretch were removed, since they likely were PCR artifacts.

Note that with this definition multiple valid reads could be extracted from a single PacBio read. Furthermore, valid reads were demultiplexed and trimmed to remove adapters, poly-A tail and the GGG sequence from template switching.

Retained reads were then aligned to the mm10 mouse genome with GMAP (version 2014-10-22) and only uniquely aligned molecules were kept [29]. GMAP created many gaps in the alignment. To fill these gaps and simplify the analysis GMAP blocks less than 40 bp apart were concatenated. The aligned reads were annotated with RefSeq for genes and exons. Only the RefSeq annotated isoform with highest number of exons were used for each gene. However, in rare cases we could identify exon isoforms in our sequencing data absent in RefSeq, for example *Mobp* (Additional file 2: Figure S5G-H). The number of transcript molecules and the loss of transcripts in each step of the analysis are shown in Additional file 2: Table S1 and Additional file 3: Table S2.

### ERCC alignment

For one ERCC RNA, the alignment was imperfect at the edges of the ERCC transcript. ERCC-00074 had some 3′ end transcripts at the correct position and some 3′ end transcripts 8 bp upstream from the annotated position. This consistent discrepancy was not found for other ERCC transcripts, and our interpretation of this was that there existed multiple variants of ERCC-00074. To remedy this ERCC-00074 was removed.

### Transcript identification

Each read had a Unique Molecular Identifier (UMI) and this was used to combine reads with the same UMI in the following way: If two or more reads had the same UMI and the same starting and ending positions for each exon, only one read was kept. If they differed in their most 5′ position and all reads with the same UMI had the same first 8 bases after the UMI, then only the most 5 prime position was used. In all other cases the median starting or ending position were used. The resulting combined UMIs and singleton UMI reads are hereafter called “transcripts”.

### Isoform identification

Both the variation in ERCC transcripts 5′ and 3′ ends and the within UMI variation indicated that 5′ and 3′ positions of transcripts were unreliable in the range of ±1 base pair, and that minor variations existed up to 5 base pairs. Some of this variation was probably due to variations in the ERCC spike-ins (e.g., differences in exact TSS during in vitro transcription), while the rest was due to technical bias during amplification and sequencing. Based on this information transcripts were combined into putative isoforms in the following way: all possible 5′ start sites and 3′ end sites were collected into a database; if the 5′ side of a transcript started within 5 bp from another transcript they were considered to have the same starting position and that starting position had a range spanning between those two individual starting positions. If another transcript had a starting position starting within 5 bp from any of the first two starting positions, then that position was considered to be part of the range and the range was extended. In this way, all possible 5′ starting positions were decided and analogously all 3′ ending positions were decided in the same way. All transcripts were then mapped back on this new database with starting and ending positions. If two transcripts differed in their 5′ side and/or their 3′ side they were still considered to be the same isoform if they were part of the same 5′ and 3′ range, and had the same number and starting and ending position of all other exons. In Fig. 1d and Additional file 1: Figure S5A, C, E, G, I such isoforms are shown.

As seen in Fig. 1c and d some transcripts in the data set are subject to degradation or alternatively that polymerase didn’t reach full length for all transcripts, both of which leads to shorter transcripts on the 5′ side. In order to take that into account a set of “Conservative isoforms” was created were only transcripts that included the annotated 5′ first exon were retained. As seen in Additional file 1: Figure S3 and S8 the within-UMI variance in exon-junctions were on the same order of magnitude as the variance in coding exon-junctions. For “conservative isoforms” exon junction’s start and end positions with 1 bp difference were not considered a separate isoform.

In Fig. 2a-d an even more conservative isoform set is used, where only differences in exon cassette usage are considered alternative isoforms.

### Calculation of offset from median

Only transcripts mapping to previously known multiexon isoforms were used. For a certain gene and exon structure the median and the deviation from median among all transcripts was calculated for the 5′ position, each exon-exon junction start and end position and the 3′ position. Note that only exons structures to which 2 or more transcripts mapped were used. Similarly, the offset from median was calculated for reads with the same UMI.

### Isoform events

Isoform events were divided into “Exon position”, TTS, TSS and “Exon cassette inclusion”. An exon cassette inclusion event is an event where a gene has more than one exon structure. E.g. 1-2-3-4 would be one exon structure, and 1-3-4 another. In the case a gene has only those 2 exon-structures the number of events would be 1, since only exon-structures apart from the first per gene was counted. An exon position event is an event where a transcript with a specific exon-structure has an exon-junction with a different start or end position than another transcript with the same exon-structure and the gene. E.g. two transcripts mapping to the same gene with exon-structure 1-3-5-6 have a different end position for exon 3. Here also only events beyond the first event per gene are counted. A TSS event is when two transcripts mapping to a gene with the same first exon has two different starting positions that are not part of the same starting-position cluster. A TTS event is when two transcripts mapping to gene with the same last exon has two different ending positions that are not part of the same ending-position cluster.

### Coding and non-coding transcripts

Each exon’s start and end-position for each transcript were annotated as coding or noncoding according to the Consensus CDS, CCDS.20150730 [30], see Additional file 4. A gene with two isoforms can differ either in its coding and non-coding part, or only in one of them, so the total number of coding and non-coding isoforms will be greater than the total number of isoforms. A number of transcript’s 3′ and 5′ ends were classified as coding. We presume this is an artifact and therefore the 3′ and 5′ coding ends were excluded from the analysis and Fig. 3a.

### Statistical analysis of coding vs. non-coding transcripts

The divergence from the median at 5′, 3′, and exon junctions for UMIs mapping to different exon-structures wasn’t normally distributed (see Additional file 1: Figure S3), so to examine the difference between coding and non-coding sites a two-sided non-parametric Wilcoxon rank signed test was used.

### Isoforms per gene extrapolation

Preseq was used to extrapolate the number of isoforms per gene. Preseq was originally intended to measure the complexity of libraries from the number of unique positions and read depth and to get an estimation of how deep the library would need to be sequenced in order to reach the intended coverage [27]. Here Preseq was instead used to get an estimation of total number of isoforms per cell, and this number was then divided by total number of genes expressed in each single cell to get the number of isoforms per gene and cell. The output from Preseq shows an extrapolation of how the number of isoforms grows with increased number of sequenced transcripts. To be able to compare between cells with different read depth the number of putative isoforms were normalized to the number of genes sequenced per sample.

### Validation of PacBio sequencing with Sanger sequencing

To validate that the findings from PacBio sequencing wasn’t an artifact from the sequencing reaction another sequencing method, Sanger sequencing, was used.

Primers were designed with Primer3 software [31]. Specificity of the primers were validated with primer blast [32]. Targeted PCR was performed for 5 cells with a total of 23 primer pairs, as shown in Additional file 1: Figure S5, with cDNA for two previously PacBio sequenced single cells (oligo 1.1 and oligo 1.2). PCR reaction was performed with Kapa HiFi Hotstart PCR kit and the cycling temperatures were: 95 °C 5 min, the 35 cycles of 98 °C – 20 s, 60 °C – 15 s and 72 °C – 60 s, followed by 5 min extension at 72 °C. PCR products were purified with the QIAquick PCR purification kit (Qiagen) according to manufacturer’s instruction, and the applied to gel electrophoresis using Invitrogen’s E-gel system. PCR products forming distinct band in the gel were dissected out and purified using QIAquick gel extraction kit (Qiagen) according to manufacturer’s instruction, and sent for Sanger sequencing at Eurofins Genomics.

## Additional files

Additional file 1: Contains supplemental information. (PDF 10883 kb)
Additional file 2: Contains a table with a summary of the long and short data sets. (XLSX 61 kb)
Additional file 3: Contains a table with a summary of the six sequenced cDNA libraries and a comparison with the previously sequenced STRT libraries. (XLSX 59 kb)
Additional file 4: Contains all isoforms. (XLSX 793 kb)
Additional file 5: Is a summary of additional file 4 that contains all genes, number of isoforms per sample and number of coding isoforms. (XLSX 177 kb)

## Abbreviations

ERCC: External RNA controls consortium
Mbp: Myelin basic protein
Oligo1: Oligodendrocyte type 1 (immature oligodendrocyte)
Oligo5: Oligodendrocyte type 5 (mature myelinating oligodendrocyte)
OPC: Oligodendrocyte precursor cell
PacBio: Pacific bioscience
SMRT cell: Single molecule real-time
STRT: Single-cell tagged reverse transcription
TSS: Transcription start site
TTS: Transcription termination site
UMI: Unique molecular identifier
VLMC: Vascular and leptomeningeal cells
ZMW: Zero-mode waveguide
