# Supplementary material for: Single-cell mRNA isoform diversity in the mouse brain
Source: BMC Genomics. 2017 Feb 3;18:126. doi: 10.1186/s12864-017-3528-6 (PMC5291953; doi:10.1186/s12864-017-3528-6)
Supplement: Additional file 1: — Contains supplemental information. (PDF 10883 kb) [file 12864_2017_3528_MOESM1_ESM.pdf]

Additional File 1

Figure S1 – Quality of PacBio Reads
Figure S2 – Comparison Long and Short data sets
Figure S3 – Within transcript variation
Figure S4 – No increased bias for longer ERCC transcript
Figure S5 – Verification of isoforms by Sanger and amplification-free PacBio seuqencing
Figure S6 – Major isoform expression
Figure S7 – Coding and non-coding variation
Figure S8 – Example ideal read
Figure S9 – Example concatenated read
Figure S10 – List of custom oligonucleotides

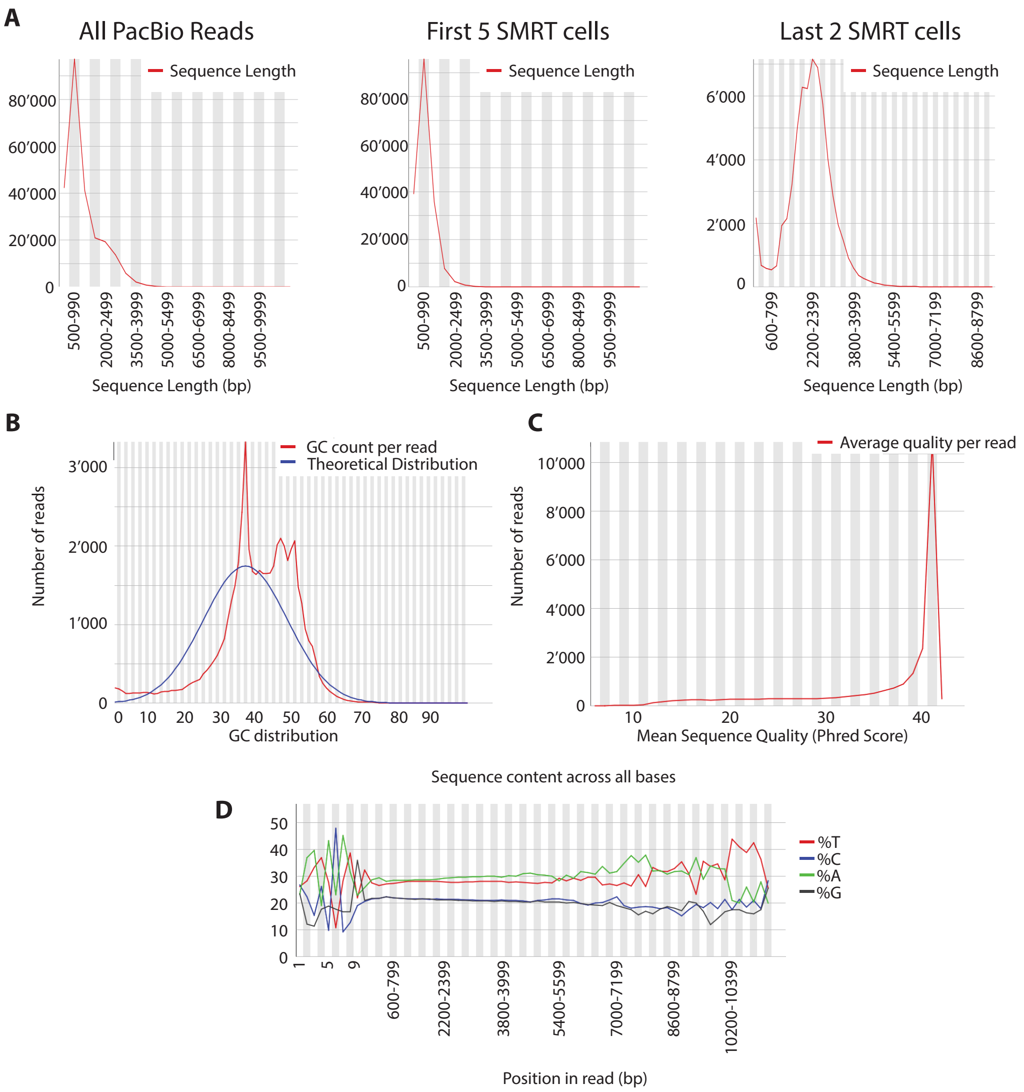

Figure S1

(A) Quality of PacBio reads. Comparison of read length between the first 5 SMRT cells and the last 2 SMRT cells. As mentioned in Methods there was an added purification step and a change in chemistry between the first five SMRT cells and the last two. Note the change of scales on the vertical axis for the last two SMRT cells. (B) GC distribution among all pooled PacBio sequences. The theoretical distribution was calculated from the observed data and used to build a reference distribution. (C) Average quality per read. (D) Sequence nucleotide content across all bases. Note the shift in scale in the X-axis to allow for individual bases at the start of the sequence. Note also that few reads are longer than 5 kb. All subfigures in Figure S1 were made with the software FastQC

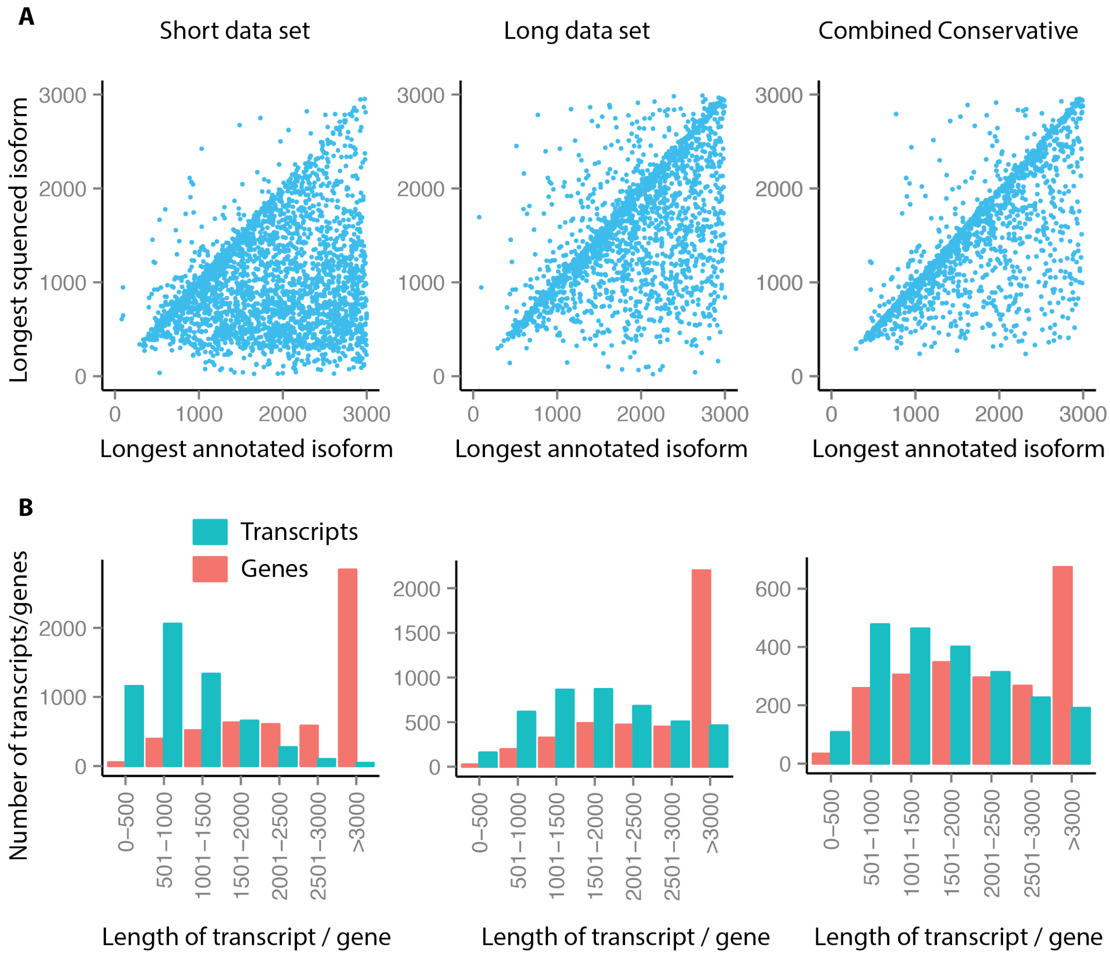

Figure S2
Comparison between annotated and sequenced gene length for the short and long data set as well as the conservative data set (see Methods), which contains reads from both the long and short data set. (A) Scatterplot depicting for each gene the longest sequenced transcript and the longest annotated isoform in bp. (B) Distribution of transcript lengths. The green bars show number of observed transcripts, while the red bars shows actual annotated lengths of the corresponding genes.

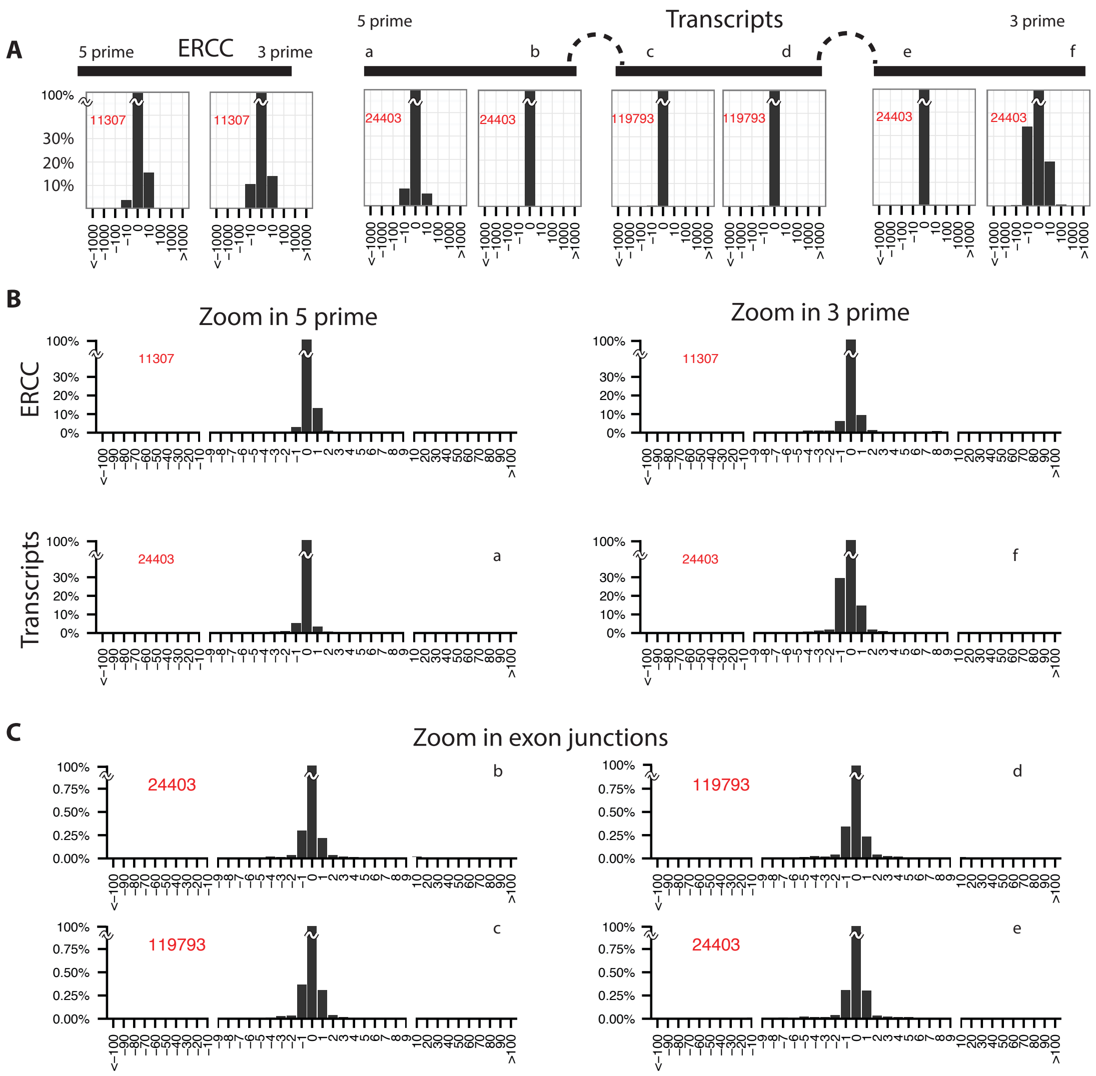

Figure S3
Within-transcript variation based on reads sharing the same UMI, showing technical errors in isoform boundaries. (A) Histograms showing the offset from median alignment position for 5′ ends, 3′ ends and splice sites. (B) Magnified view of 5′ and 3′ ends. (C) Magnified view of exon junctions.

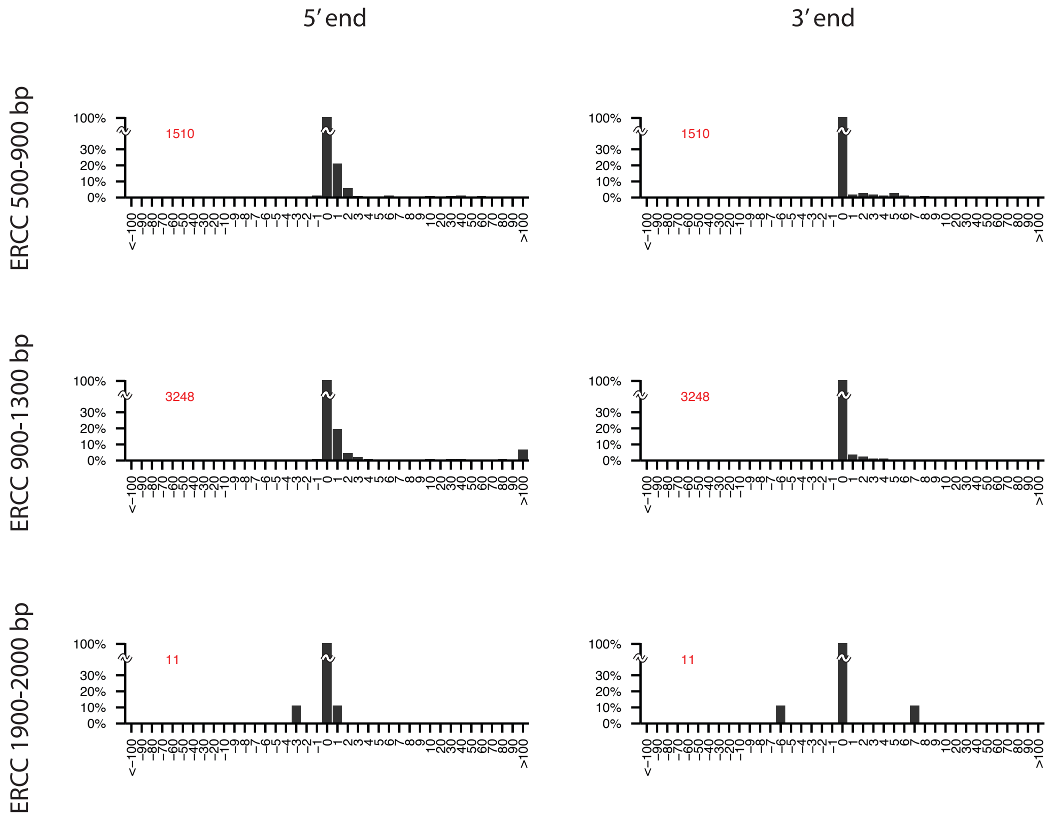

Figure S4
Histogram showing the offset from median for 5′ , 3′ ends and splice sites, in terms of percentage of zero offset for reads mapping to ERCC, split by ERCC length. Red, observed number of transcripts. Note that the Y-axis is truncated and that the X-axis is shown in bins of 10 bp outside the region of ± 10 bp. Note also that there were no ERCC transcripts with length between 1300 and 1900 bp and that the number of transcripts longer than 1900 bp are very few.

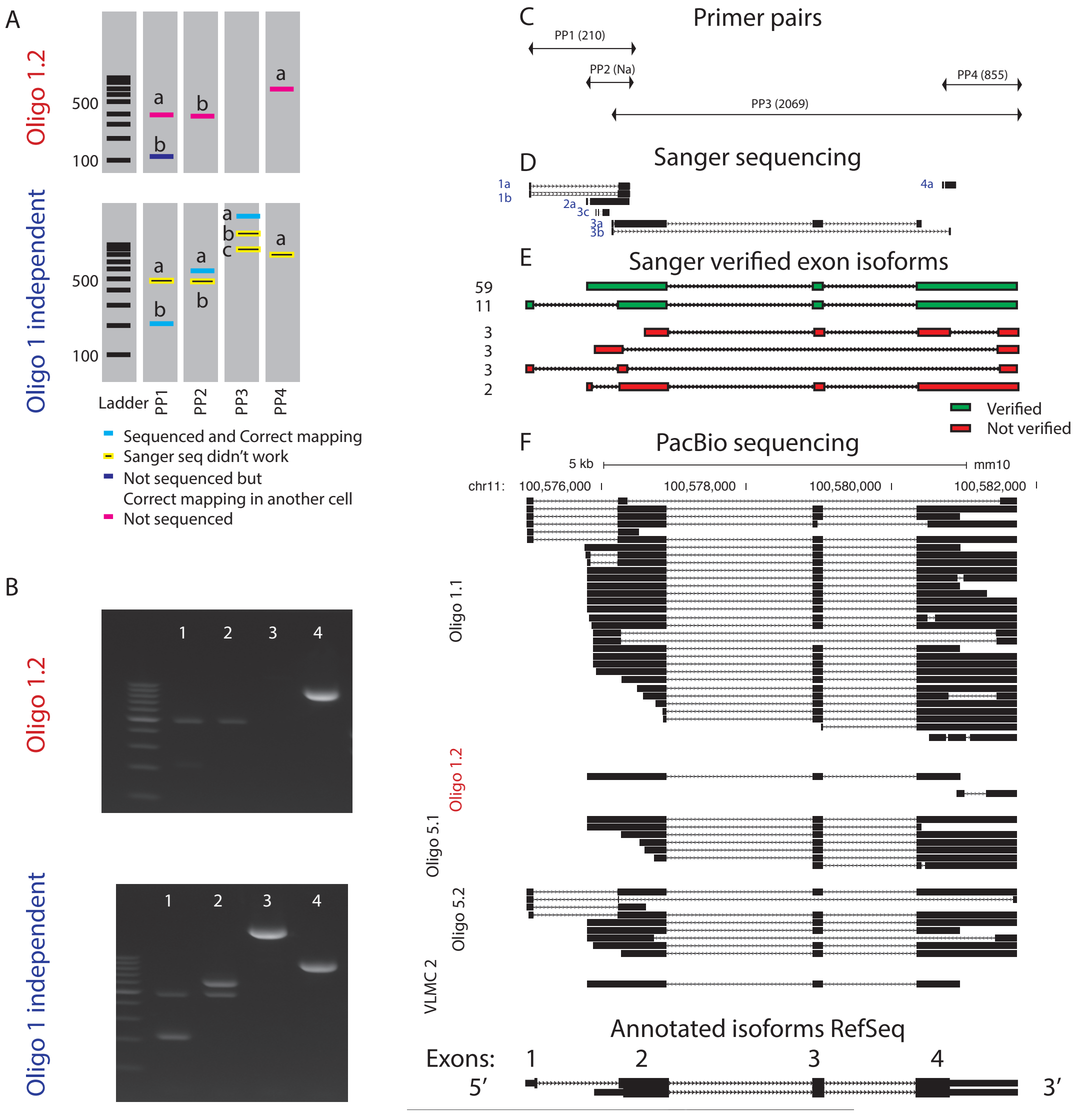

Figure S5A

Sanger Verification of *CNP* (2',3'-Cyclic Nucleotide 3' Phosphodiesterase)

A| Simplification of gel images. Each band is annotated with a letter and the corresponding alignment of Sanger sequences are shown in C. Each band is also annotated for if the it was Sanger sequenced and if the Sanger sequence mapped to the correct position in the genome. For Oligo 1.1, Oligo 5.1, Oligo 5.2 and VLMC 2 Pacbio single-cell data exists (marked in black). For cell Oligo 1.2 Pacbio and limited Sanger sequencing exists (marked in red). Additionally Sanger sequencing was done on a cell not sequenced by Pacbio (Oligo 1 external cell, marked in blue)

B| Gel visualization of PCR products

C| Genomic position of the different primerpairs. The number in brackets is the expected PCR product length according to according to NCBI's Primer-Blast.

D| Sanger sequencing results mapped to the mouse genome

E| Isoforms verified by Sanger sequencing:

- PP1 verified that exon 1 isn't an artifact and that when exon 1 is prescent then exon 2 is truncated
- PP2 verified that full length exon 2 exists, but failed to show internal introns in exon 2 that could be seen in the sequencing data
- PP3 verified isoforms with exon 3 exists but failed to show isoforms without exon 3
- PP4 failed to verify exon 4 internal introns.

The number to the left of each isoform indicate how many instances of that exon isoform that were sequenced in all single cells combined. Note that most sequences comes from Oligo 1.1, and that sanger sequencing was done on an independent cell.

F| Single cell isoforms from PacBio sequencing

Na, Not applicable, i.e. no valid primer product in primer blast

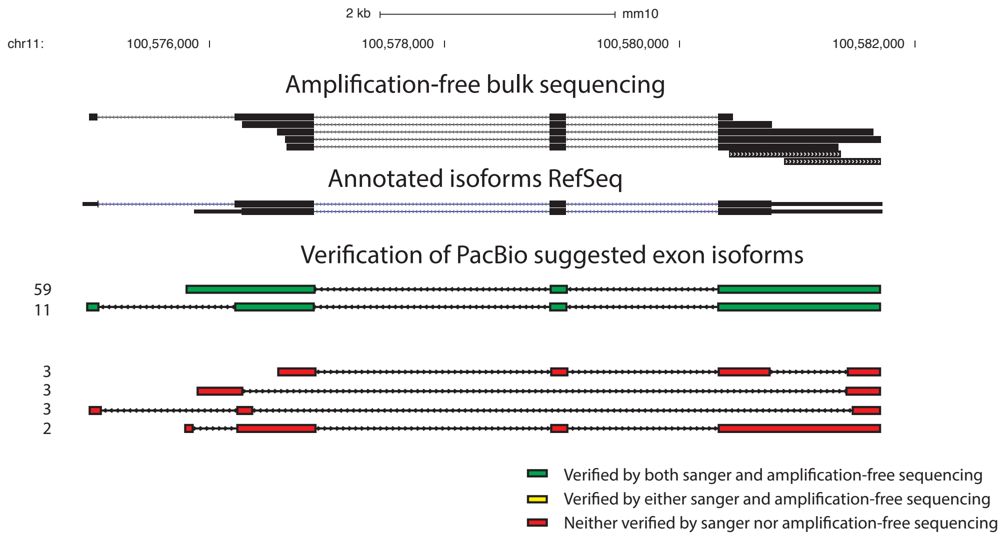

Figure S5B

Amplification-free PacBio sequencing of *CNP* (2',3'-Cyclic Nucleotide 3' Phosphodiesterase)

The single cell PacBio exon isoforms that are verified by both sanger sequencing and amplification-free bulk sequencing are colored green. If either sanger sequencing or bulk sequencing could verify an isoform it is colored yellow. If no method could verify the isoform it is colored red.

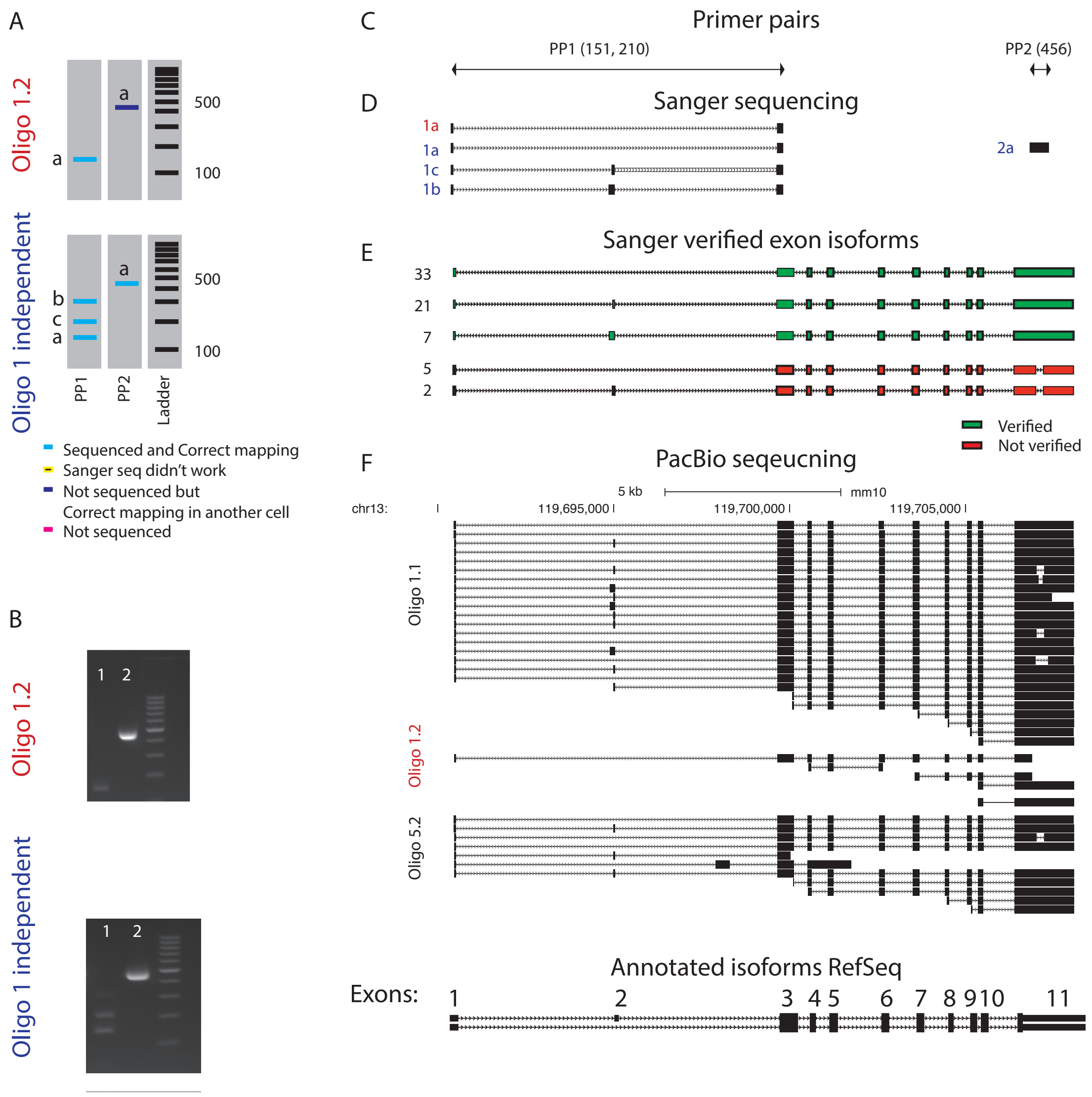

Figure S5C

Sanger Verification *Hmgcs1* (3-Hydroxy-3-Methylglutaryl-CoA Synthase 1)

A| Simplification of gel images. Each band is annotated with a letter and the corresponding alignment of Sanger sequences are shown in C. Each band is also annotated for if the it was Sanger sequenced and if the Sanger sequence mapped to the correct position in the genome. For Oligo 1.1 and Oligo 5.2 Pacbio single-cell data exists (marked in black). For cell Oligo 1.2 Pacbio and limited Sanger sequencing exists (marked in red). Additionally Sanger sequencing was done on a cell not sequenced by Pacbio (Oligo 1 external cell, marked in blue)

B| Gel visualization of PCR products

C| Genomic position of the different primerpairs. The number in brackets is the expected PCR product length according to according to NCBIs Primer-Blast.

D| Sanger sequencing results mapped to the mouse genome

E| Isoforms verified by Sanger sequencing:

- PP1 verified the existence of three isoforms of exon 2, either it is absent, or it has a short or long version of the exon.
- PP2 failed to verify internal introns in exon 11

The number to the left of each isoform indicate how many instances of that exon isoform that were sequenced in all single cells combined.

F| Single cell isoforms from PacBio sequencing

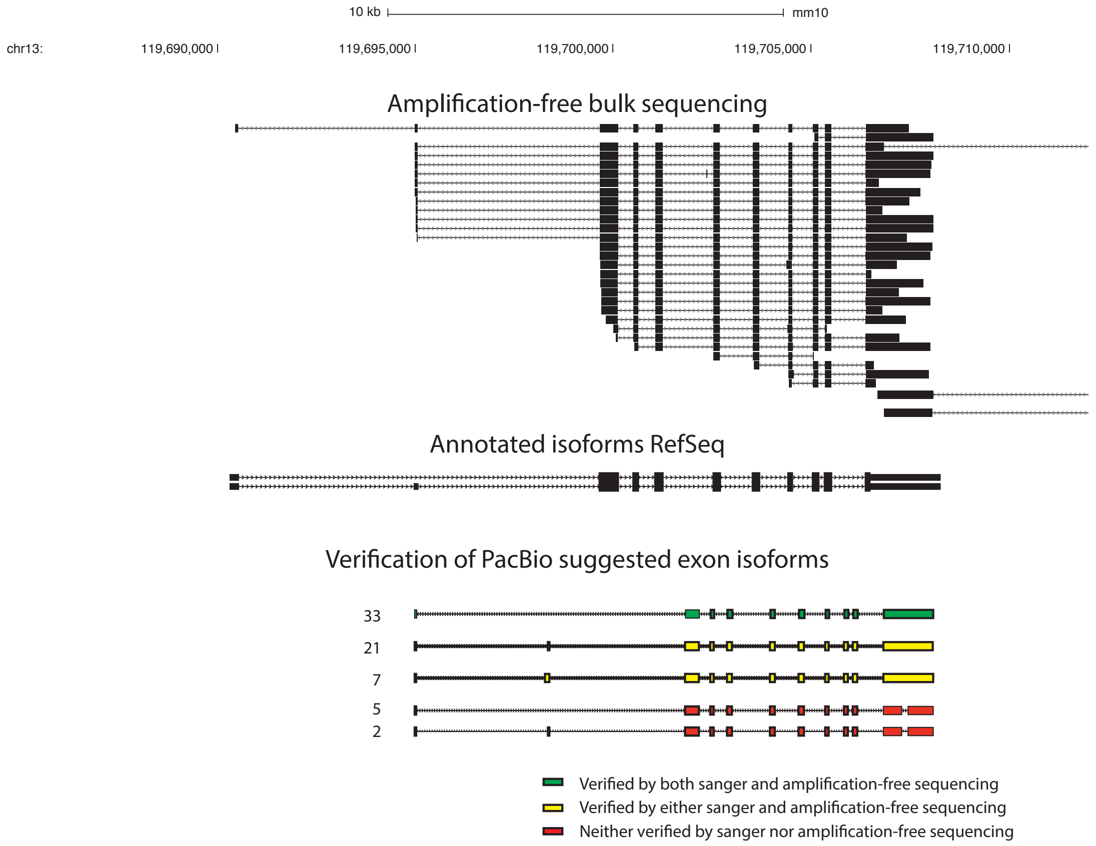

Figure S5D

Amplification-free PacBio sequencing of *Hmgcs1* (3-Hydroxy-3-Methylglutaryl-CoA Synthase 1)

The single cell PacBio exon isoforms that are verified by both sanger sequencing and amplification-free bulk sequencing are colored green. If either sanger sequencing or bulk sequencing could verify an isoform it is colored yellow. If no method could verify the isoform it is colored red.

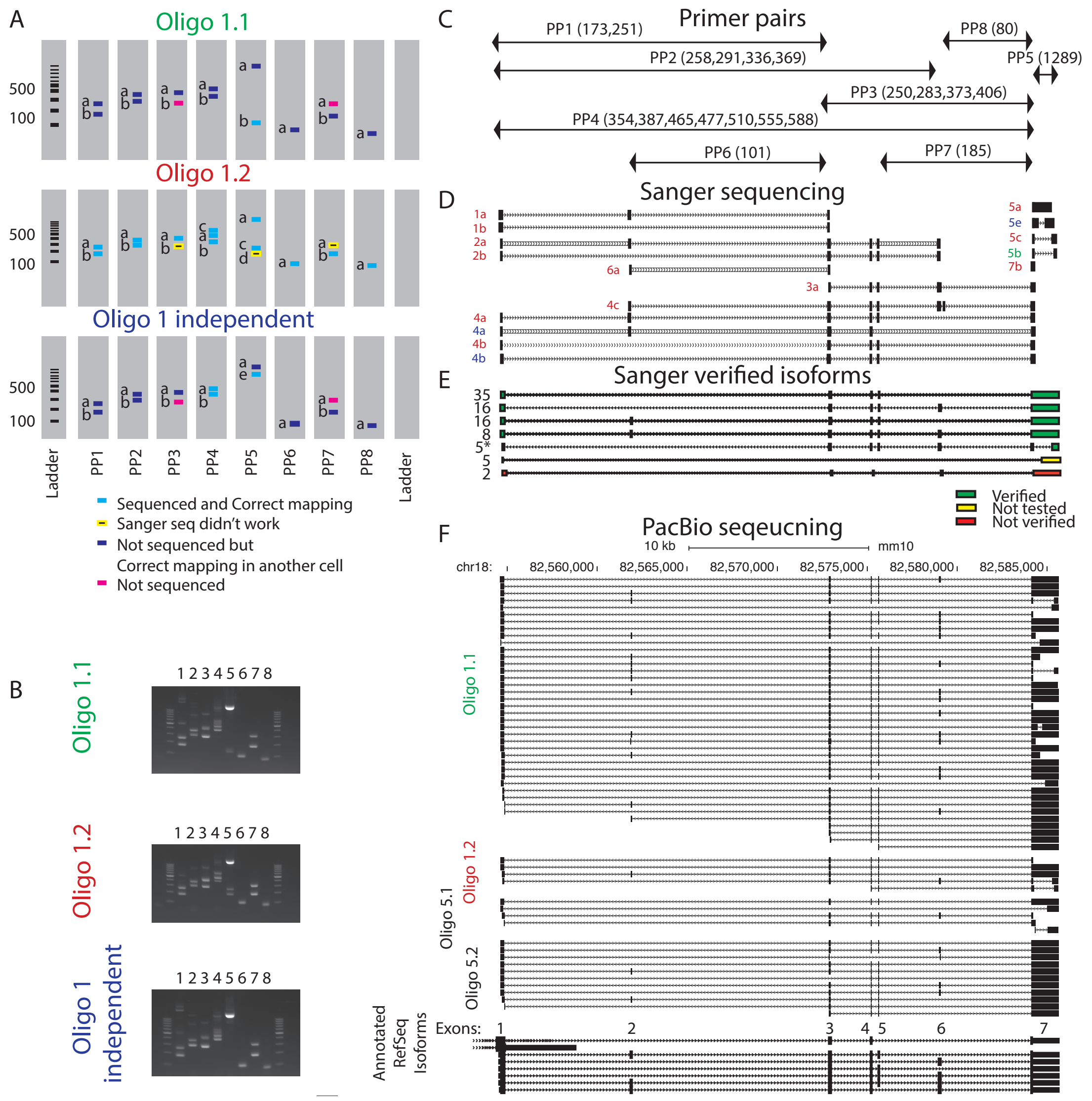

Figure S5E

Sanger Verification *Mbp* (Myelin Basic Protein)

A| Simplification of gel images. Each band is annotated with a letter and the corresponding alignment of Sanger sequences are shown in C. Each band is also annotated for if the it was Sanger sequenced and if the Sanger sequence mapped to the correct position in the genome. For Oligo 1.1 Pacbio sequencing, PCR amplification and limited Sanger sequencing data exists (marked in green). For Oligo 1.2 Pacbio and Sanger sequencing exists (marked in red). For Oligo 5.1 and Oligo 5.2 Pacbio single-cell data exists (marked in black). Additionally Sanger sequencing was done on a cell not sequenced by Pacbio (Oligo 1 external cell, marked in blue)

B| Gel visualization of PCR products

C| Genomic position of the different primerpairs. The number in brackets is the expected PCR product length according to according to NCBI's Primer-Blast. Note that PP6, PP7 and PP8 are spanning exon junctions.

D| Sanger sequencing results mapped to the mouse genome

E| Isoforms verified by Sanger sequencing:

- PP1 and PP6 verifies both isoforms of exon 2. (Note PP6 upstream primer spans the junction between exon 2 and 3)
- PP2 also verifies both isoforms of exon 2 and that exon 3, 4 and 5 are expressed together.
- PP3 verified one of the isoforms with exon 6, however the isoform without exon 6 could be seen by gel electrophoresis but not be verified by Sanger sequencing
- PP4 verifies both the existence of 2 isoforms of exon 2 and 2 isoforms of exon 6
- PP5 verified the existence of internal introns in exon 7, and that those isoforms varies from cell to cell.
- PP7 verified the connection between exon 5 and 7 (note that the upstream primer spans the exon junction between exon 5 and 7).
- PP8 failed to map. (Note that the downstream primer spans the exon junction between exon 6 and 7).

The number to the left of each isoform indicate how many instances of that exon isoform that were sequenced in all single cells combined. The (*) indicated all isoforms with internal intron in exon 7.

Note that not all exon isoforms were attempted to be verified by Sanger sequencing, for example the isoform spanning only exon 1 and 7.

F| Single cell isoforms from PacBio sequencing

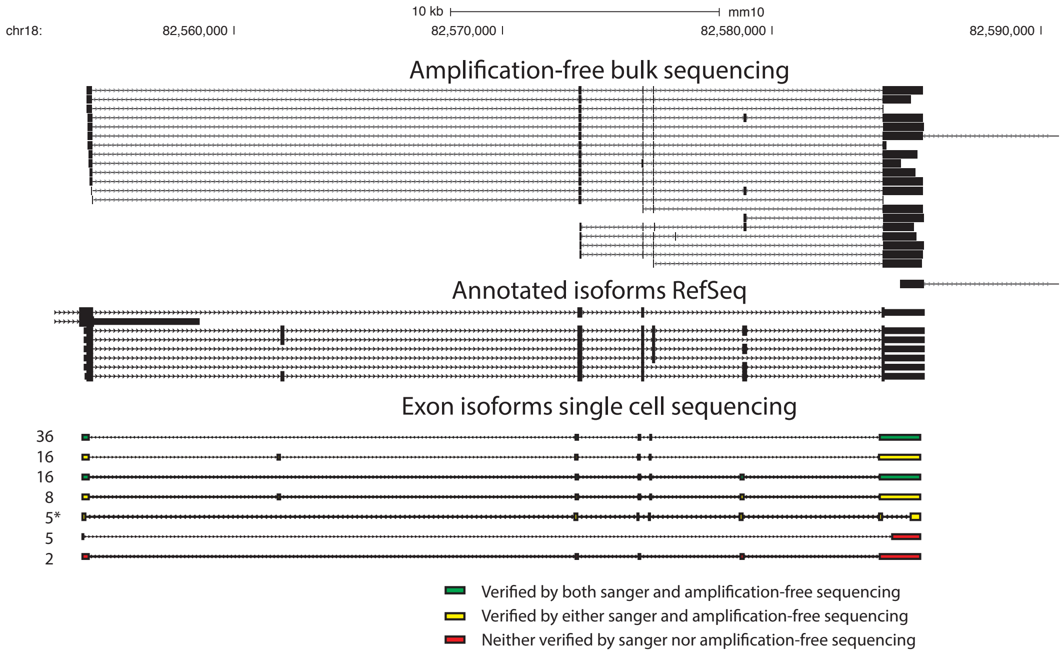

Figure S5F
Amplification-free PacBio sequencing of *Mbp* (Myelin Basic Protein)

The single cell PacBio exon isoforms that are verified by both sanger sequencing and amplification-free bulk sequencing are colored green. If either sanger sequencing or bulk sequencing could verify an isoform it is colored yellow. If no method could verify the isoform it is colored red. The star denotes that all isoforms with 3′ UTR introns are calculated, regardless of other exon structures and the length of the intron.

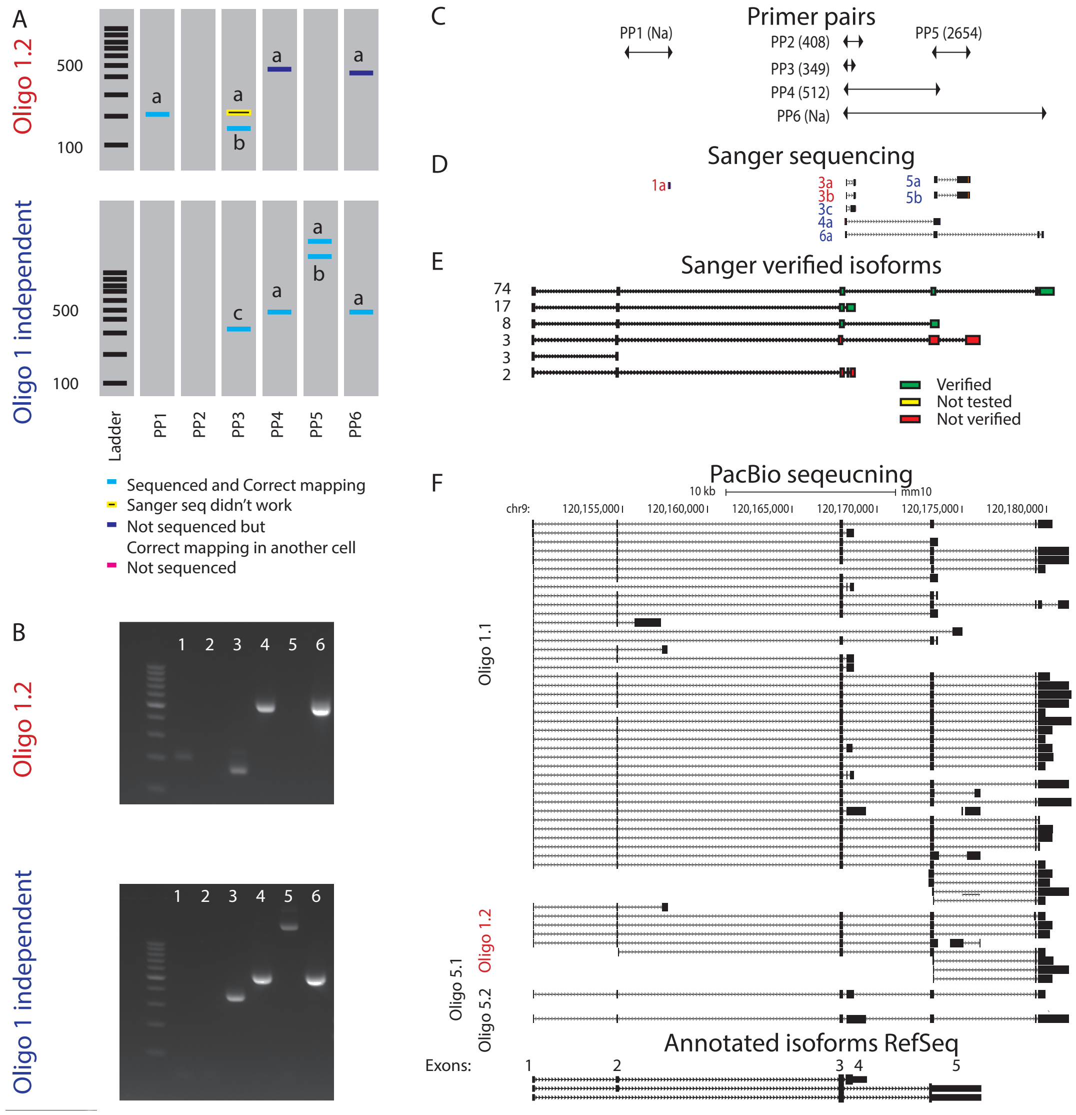

Figure S5G

Sanger Verification *Mobp* (Myelin-Associated Oligodendrocyte Basic Protein)

A| Simplification of gel images. Each band is annotated with a letter and the corresponding alignment of Sanger sequences are shown in C. Each band is also annotated for if the it was Sanger sequenced and if the Sanger sequence mapped to the correct position in the genome. For Oligo 1.1, Oligo 5.1 and Oligo 5.2 Pacbio single-cell data exists (marked in black). For cell Oligo 1.2 Pacbio and limited Sanger sequencing exists (marked in red). Additionally Sanger sequencing was done on a cell not sequenced by Pacbio (Oligo 1 external cell, marked in blue)

B| Gel visualization of PCR products

C| Genomic position of the different primerpairs. The number in brackets is the expected PCR product length according to according to NCBI's Primer-Blast.

D| Sanger sequencing results mapped to the mouse genome

E| Isoforms verified by Sanger sequencing:

- PP1 verifies the existence of an exon between exon 2 and 3. Note that the forward Sanger sequencing primer is on exon 2.
- PP2 didn't work
- PP3 verifies two different lengths of exon 4
- PP4 verifies a long version of exon 5
- PP5 verifies an internal intron in exon 5
- PP6 verifies that if exon 5 is short if exon 6 and 7 exist

The number to the left of each isoform indicate how many instances of that exon isoform that were sequenced in all single cells combined. Due to space limitation only exon isoforms with more than 1 UMI are shown.

F| Single cell isoforms from PacBio sequencing

Na, Not applicable, i.e. no valid primer product in primer blast

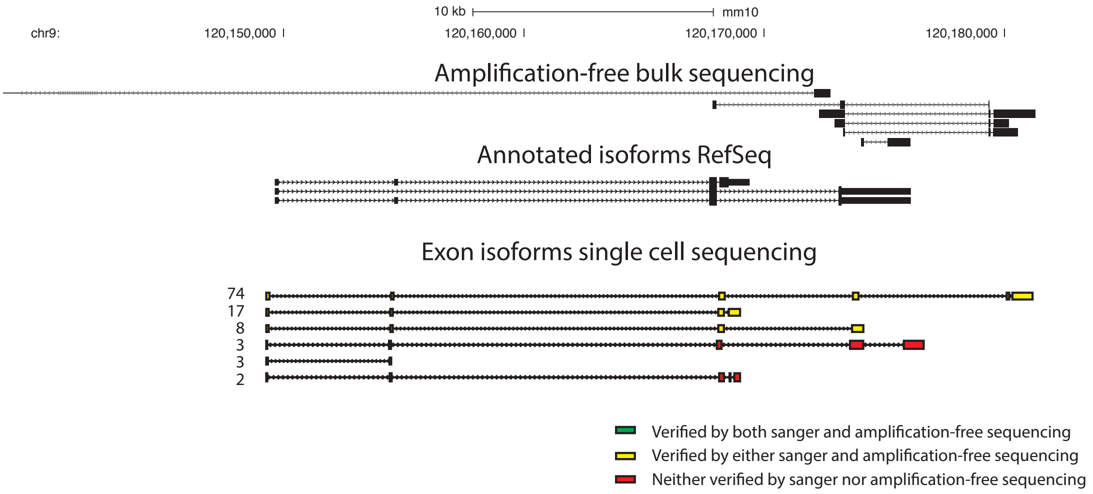

Figure S5H

Amplification-free PacBio sequencing of *Mobp* (Myelin-Associated Oligodendrocyte Basic Protein)

The single cell PacBio exon isoforms that are verified by both sanger sequencing and amplification-free bulk sequencing are colored green. If either sanger sequencing or bulk sequencing could verify an isoform it is colored yellow. If no method could verify the isoform it is colored red.

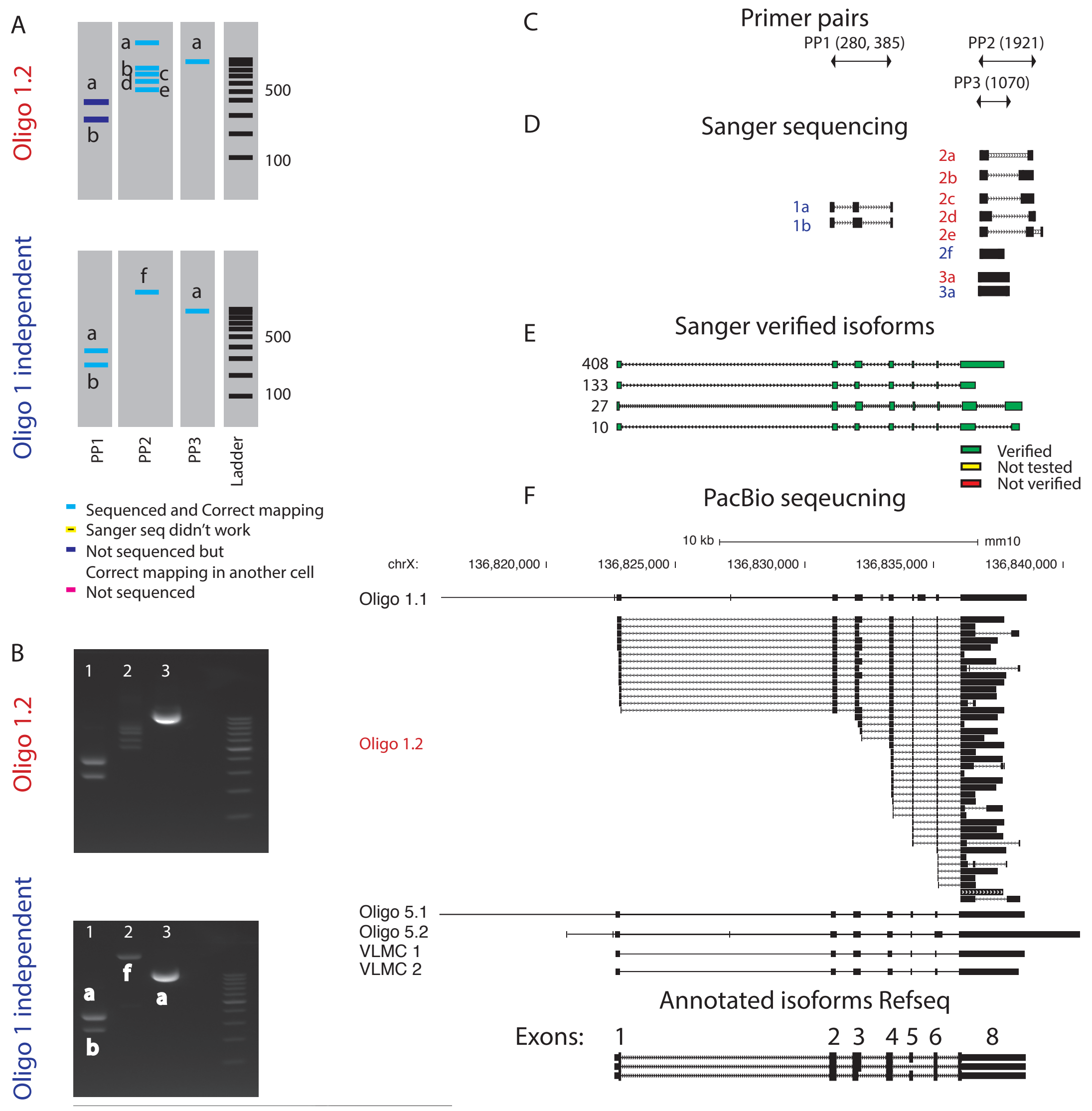

Figure S5I

Sanger Verification *Plp1* (Proteolipid Protein 1)

A| Simplification of gel images. Each band is annotated with a letter and the corresponding alignment of Sanger sequences are shown in C. Each band is also annotated for if the it was Sanger sequenced and if the Sanger sequence mapped to the correct position in the genome. For all cells except Oligo 1.2 only Pacbio sequencing data exists (marked in black). For cell Oligo 1.2 Pacbio and limited Sanger sequencing exists (marked in red). Additionally Sanger sequencing was done on a cell not sequenced by Pacbio (Oligo 1 external cell, marked in blue)

B| Gel visualization of PCR products

C| Genomic position of the different primerpairs. The number in brackets is the expected PCR product length according to according to NCBI's Primer-Blast.

D| Sanger sequencing results mapped to the mouse genome

E| Isoforms verified by Sanger sequencing:

- PP1 verified two isoforms of exon 3
- PP2 and PP3 verified multiple internal intron of exon 8

The number to the left of each isoform indicate how many instances of that exon isoform that were sequenced in all single cells combined. Due to space limitation only exon isoforms with more than 1 UMI are shown.

F| Single-cell isoforms from Pacbio sequencing. Note that for all cells except Oligo 1.2 the Pacbio sequencing isoforms have been collapsed due to space limitations.

..

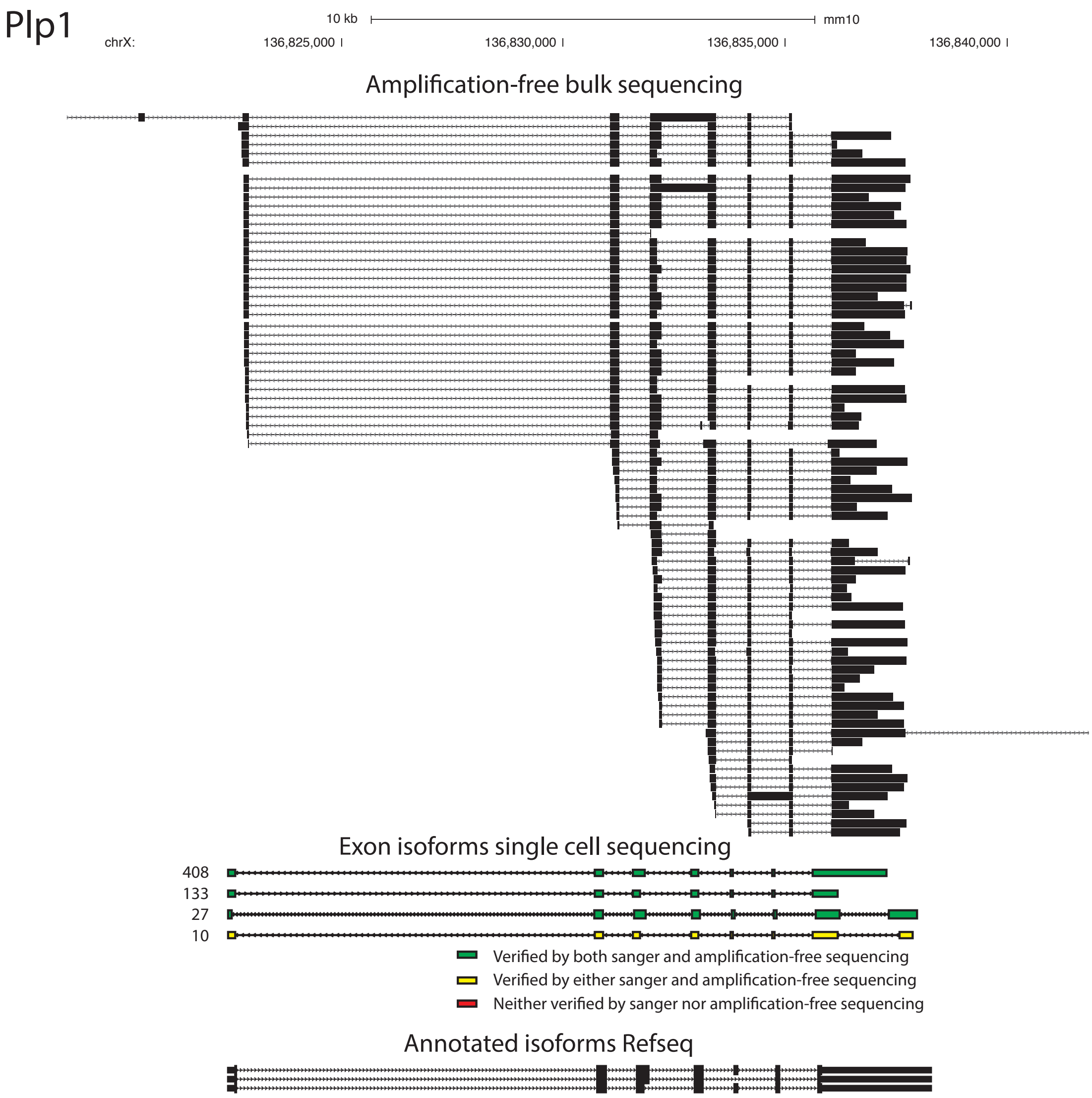

Figure S5J

Amplification-free PacBio sequencing of *Plp1* (Proteolipid Protein 1)

The single cell PacBio exon isoforms that are verified by both sanger sequencing and amplification-free bulk sequencing are colored green. If either sanger sequencing or bulk sequencing could verify an isoform it is colored yellow. If no method could verify the isoform it is colored red.

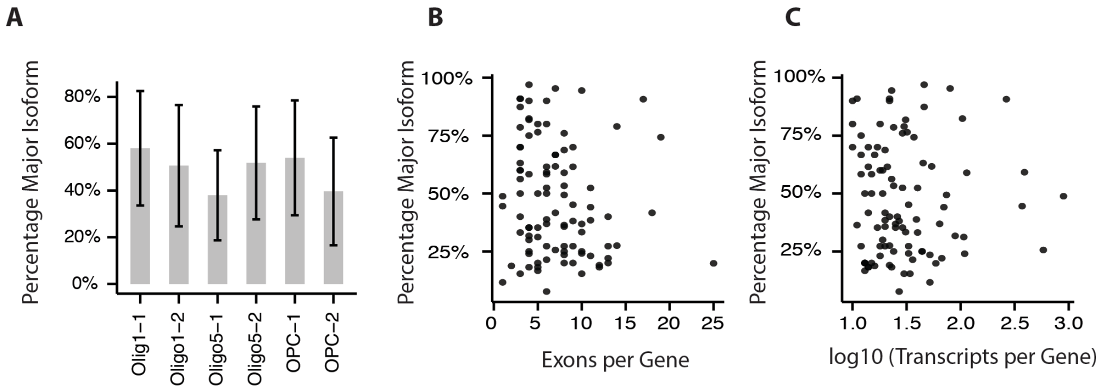

Figure S6

Major isoform usage. A) Percentage major isoform was calculated across all genes with more than 10 transcripts for each cell. The major isoform was defined per gene and cell and could therefore change between cells. B) Percentage major isoform and number of exons per gene. Each dot is a gene. C) Percentage major isoform and gene expression. Each dot is a gene.

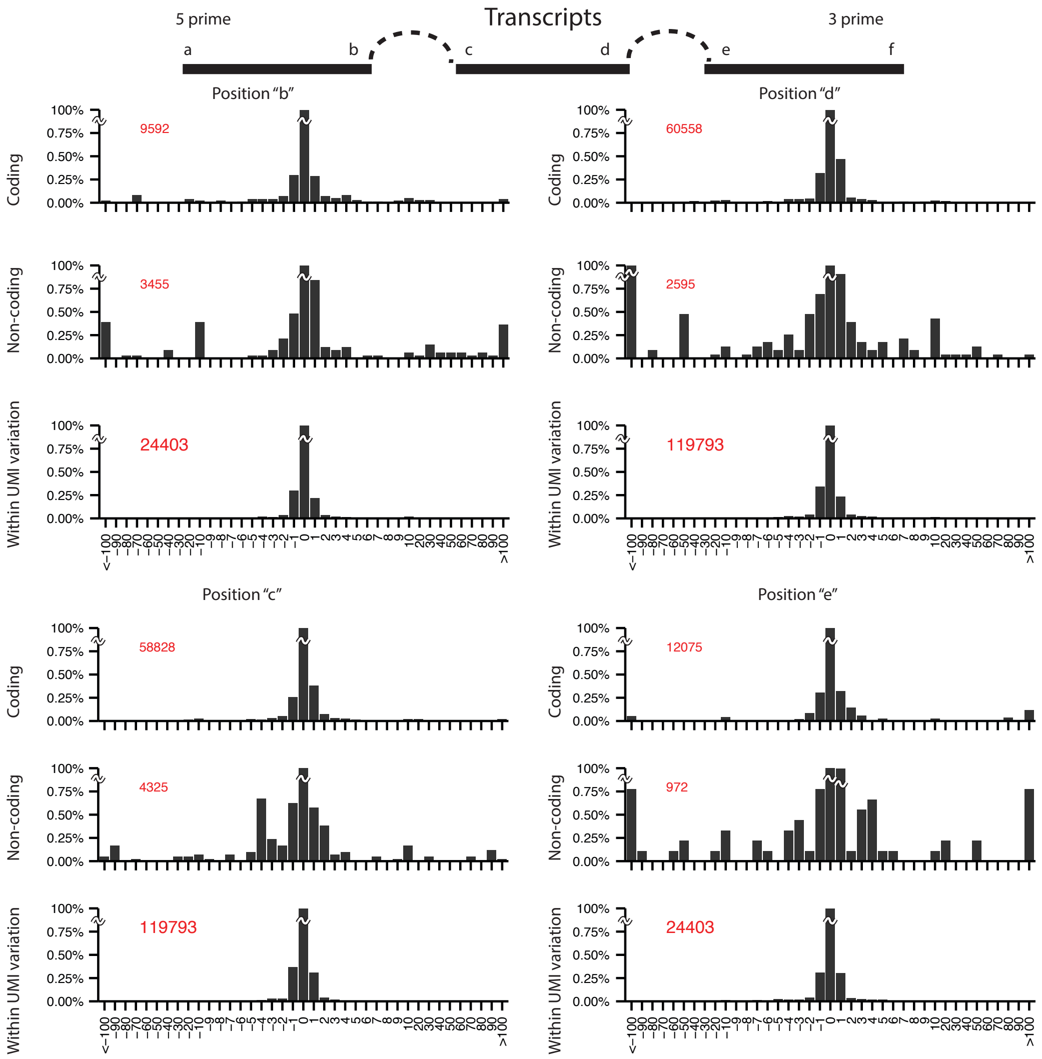

Figure S7

Zoom-in on coding and non-coding exon splice sites. Histogram showing the offset from median for splice sites, in terms of percentage of zero offset. The data for coding and non-coding region is the same as in figure 3A, but. The data for within transcript variation is the same as in Additional file 1: Figure S3, but zoomed-in. Note that the Y-axis is truncated and that the X-axis is shown in bins of 10 bp outside the region of ± 10 bp.

Figure S8: Example ideal read

>m150922_014300_42134_c100884922550000001823198604021672_s1_p0/3975/ccs
GATCTCTACTATATGCGAATGATACGGCGACCACCGATGGTATAGGGATCA
GTAGATTTGAACAGAGCCAGAGGTACCATGCTCTTGCCAGTAAAATTCTTT
TCCCATGTTTTTGAATACATTTGTATTTAATGTGGCTGAAATGACAAAAACA
AAGGTCCTAAATTTTAGAATAGATGTTATCTTTTTATTTAATCTTTTAAAAA
AAAAACTGGATGTTTCAATCTTTTAAATTGCAAGACACAAGGCAATTCCAA
CTGGGCATTTCAATCTGGTTTTAGGTGCTTTAGAGATAATTGCTAGGCAGT
GCCAAATGAAGGCATTAGTGCTTTGTACCCAGAAATTGGCCTCTACATGCA
GCACTAAGATTAATGCAGATTTCTCTTGAACCTTCCAGTCTTCCTTGTTGGT
GGTAATGTGCTTTGTTCTTTCCTTTTTTTCCTCAAGAAGAAATGTTAGTATG
TCTTAGAACCTAGGTAACGAAGTGCACTTATGCTCATAAAATTGCTTGCAC
CTAATCCAAAAGTCGTGGTCTTGCCTTAGTCCATTAACATGGTCTTCATTTC
CAGTTTGCATTGACTGCTACAGCACTTACTAATTTGGCTTTTATGTTTAAAT
GGTTCTGTGATAATCAAAACTTAAATAGTTACTGTTCGTTCATTGTGGTACT
TTTGTATTCAGTTCTGTACTCAGAGATGGCACCAATTGTTCATTTACTATGC
ACCACCTACTGCTTATATAAGAGAACTAATAACTCTTTCAGCCGATACTTA
ACACATCATGTATTCCTTGCAGCTTCTAGGACATCTTCCATACATGTATGGA
TGTGTACATACATATACATGTGTACATACTGGTATGGCCCAGCACATATTT
CTTAAGCTCTGCAGCCATATATAGAAGTCCTTGTTATTTGGGAGGACAGAT
TAAATGCCTTTCATTCACAAGAAAATGAAGAGCTCTCTGGGTTCCAAAATG
CTGATGTTGAATACATGATATATATGCACACATATAGGCACTTGCATGTTA
GCATGTTCATGTCTGTGACTGTCTTTTGAGTTATATAGGAGTGGCAGAGAT
TGTGTCACTTTAACTTGTTGTCGACTTAGCCTGAAAACCTTACTAGTTCAGA
ATCATACTCCTGACTGTCTTCTGGAGCAACAGGGAACTGCATGGCCAACTT
AAAGTACTTGTTATAAATGAACCAGAAATCTGATTTTAAAATACATTTTTCA
TTTTAATTGTTTTTGCATTTTGTTTTGTTAATCCTTAAAGGATGGATTGTGC
TTTTAAGAATGTAAGTGTTATGTATTTGTCATGAATCAACAAAACAGCTTTT
AAAAAAGACTAATTGGAACAAGTGGGTGGCACTGGCTTAATGCTGTTAATG
TTTCTAAACGATTTTACATTTAGATTATATATCGGATTCATATTGAGATACC
TCCAAAGCACTGTTTTATAGAAAGATCTGACTTCAATAAATATTTTTGTATT
TTACATGGGCCAGTTTATGTACCGCTATTTACACTATTATTTCCTATAGACA
ATACACACTGTCTTTGTAGTTTTTGTATTTTTGTTTTGTTTTACTGGTTTTGC
TTCTTGATGGTAATATACTCTGTCTGGTGTGGGATATTTTCCACACTTTAGA
ATTTGTATAAGAAACTGGTCCATGTAAGTACTTTCCATGTTTTCTCTTCAAA
TGTTTAAAGTGCTAGCTGATGTACGTACGATATCTCTTCTCAGATATTTGCC
TGTCTGTTTGCCCAAAAATTGCTTCTAAATCAATAAAGATTCTTTTATTTCT
TAAGGAAAAAAAAAAAAAAAAAAAAAAAAAAAAAAAAAAACGATCGGT
GGTCGCCGTATCATTCGCATATAGTAGAGA

Illumina adaptor
PacBio barcodes – start
PacBio barcodes – end
UMI
Site of template switching
PolyA / PolyT

Figure S9: Example concatenated read

>m150920_000225_42134_c100885002550000001823198604021652_s1_p0/1024/ccs

TACTAGAGTAGCACTCGAATGATACGGCGACCACCGATGGAAGAGGGTGTCCTGACTTCC
TTTGATGATGAACAGTGATGTGAAAGTGTAAGCCAAACCTTTTCCTCCTCAACTTCCTTTTG
GTCATGGTTTTTCGTCACAGCAATAGAAACCCTAATTAGGATATATCCCTGAAACTGGAGTT
ACAGAAGCTTGTGAGTGGCCATGTGGTGCCAGGAATACAACCCAGCTCCTCCAGAAGGGCA
GCCAGTGCACTTAACCACGGAGCCATCTCTCCAGCCCTAGTTAACCTTTAAAAGTAATACAT
TACTATTTACAAATTAGTATATGTCAAACATTTTCTTCCTCATGTTGAAATGAAATTCTATTC
TTCCTGTGATCTTGTTTGTGTATTCTTTGGATGTTATAACTTGCCAAAAATCCTTGATATTAT
AGCTTGCCAATGTTTGCTGACAAATGTAATTCTTTAGAAAAGTTTGTTTTTCATAAACATAT
TTAGTTTTATTGTAATATTTATCTGACCAAAAAAAAAAAAAAAAAAAAAAAAAAAAA**CGA**
TCGGTGGTCGCCGTATCATTCGAGTGCTACTCTAGTATACTAGAGTAGCACTCGAATGAT
ACGGCGACCACCGATCTTGCAGGGACCGTCGGGGCTTCCTCGACGAGGCCGTTCGGAAGG
TCTCCTGCTCCGTCTCGAGAGCTGCTTTCTCCTTCCGCACACGCTACCCGGCTGCTGCGGCC
CCAGAACGCCCGGGTGAGGAGTTGGTTGTAGTGAGCAGTTCCGATCCCTTGGGCTACCGGC
GGCGAGCGCCCGAGCCGCTCCTCCCAATGGCGAAGAAGACGTACGACCTGCTTTTCAAGCT
GCTCCTGATCGGGACTCGGGAGTGGGCAAGACCTGCGTCCTTTTTCGTTTTTCGGACGATG
CCTTCAATACCACCTTTATTTCCACCATAGGAATAGACTTTAAGATCAAAACAGTGGAACTA
CAAGGAAAGAAGATCAAGCTACAGATATGGGACACAGCAGGCCAGGAGCGATTTCACACC
ATCACAACCTCCTACTACAGAGGAGCAATGGGCATCATGCTAGTGTATGACATCACCAACG
GTAAAAGCTTTGAGAACATCAGCAAGTGGCTTAGAAACATAGATGAGCATGCCAATGAAGA
TGTGGAAAGAATGTTACTAGGGAACAAGTGTGACATGGACGACAAGAGAGTTGTACCGAA
AGGCAAAGGAGAACAGATTGCAAGGGAGCATGGTATTAGGTTTTTGAGACTAGTGCAAAA
GCAAATATAAACATCGAAAAGGCGTTCTCACATTAGCTGAAGACATCCTCCGAAAGACCCC
TGTAAAAGAACCCAACAGTGAAAACGTAGATATCAGCAGTGGAGGAGGCGTGACGGGCTG
GAAGAGCAAGTGCTGCTGAGTGCTCTCCTGTCCATCTGCTGCCATCCACCATCCGGTTCTCT
TCTTGCTGCAAAATAAAACACTCTGTCCATTTTTAACTCTAAACAGATATTTTTGTTTCTCAT
CTTAACTATTCAATCCACCTATTTTATTTGTTCTTTCATCTGTGACTGCTTGCGGACTATTAT
AATTTTCTTCAAACAAACAAACAAAAATGTATAGAGAAATCATGTCTGTGAGTTCATTTTGA
GATTTACTTGCTCACTCAGCCCTGCACTTCAGTTGTATTATAGTCCAGTTCTTATCAACATT
AAACTAGAGCAATCATTTTCAACTAAAAAAAAAAAAAAAAAAAAAAAAAAAAAAAAAAA
AAAAAAAAAAAAAAAAAAAAAAAAAAAAAAAAAAAAAAAAAAAAAAAAAAAAAAAAAA
AAAAAAAAAAAAAAAAAAAAAAAAAAAAAAAAAAAAAAAAAAAAAA**CGATCGGTGGTC**
GCCGTATCATTCGAGTGCTACTCTAGTATCAGACGATGCGTCATGAATGATACGGCGACC
ACCGATCGTTTTTTTTTTTTTTTTTTTTTTTTTTTCTTCGGCTTCCTACTACCTGTGCCTTTAA
GTACTTTTCTGTCTTCTAAAGATGCTTTGTCTTCTATTTTCCTATCTTCTCTAACAGCTTTTT
CTTCACCTTCCTGTATGTCTTCTTCGTCTTTCACTTCCATTTTCTAATCTTCTTTCTCCAAATA
CTAGCCTAACCTTTTTGTTTTTTCAAGACAGGGTTTCTCTGTGTAGCCCTGGCTGTCCTGGA
ACTCACTCTGTAGACCAGGCTGGCCTCGAACTCAGAAATCCGCCTGCCTCTGCCTCCCAAG
TGCTAGGATTAAAGGCGTGCGCCACCATCGCCCGGCTAGCCTAACACTTCCATCTTCCCTGT
TTCCAACTCATGCCTTCAAACGTACATGCGCCAGTCGAAGGATTTTTATAACGGCCGTCAAC
TTAACCTACTTTTCTCAATCTTTTAAAACTACACCTCATTTTCAAACTATTCTCTACAACTTT
ACTGTTTTAAATGCCACTTAGATTCTATTTAGGTAACCTTCGTTTTAATCTACAAGGCCGAC
CTTCAAACTAGAACCTTTTAGAACTTCACAAAACCTCCCTTTACAATCTCCTAAACTGCTCT
GGTCAGCCTCCATTATACAGTACAAATACTTTTCTCCTCATTTCCTTTTTCAAACTGTTTAAA
TAAGCCTTCATGTTATCTCTTAAGATCTTCTTAGATTATTAAGACTAAGTAATATTTCGATA
AGCTACTCTATTAGCTTAAGTTTAGAGTTCTAATTCTTTTTACTGCTCAATCTTTTTAATTAA
AAACTTATCTGCGATTTCCTCGGGCTGAGTCTCCTGCCTCACGAGCTCAGCTGTGCTGCTCT
ACGCTGCTCTGCTCTCGCTGCCTGAATGCCTCCCAACCACATCGCCCTCCAAT**ATCGGTGG**
TCGCCGTATCATTCATGACGCATCGTCTGA

Illumina adaptor
PacBio barcodes – start
PacBio barcodes – end
UMI
Site of template switching
PolyA / PolyT

Figure S10: List of custom oligonucleotides (5′ to 3′ direction):

Idx1: TCAGACGATGCGTCATGAATGATACGGCGACCACCGAT
Idx2: CTATACATGACTCTGCGAATGATACGGCGACCACCGAT
Idx3: TACTAGAGTAGCACTCGAATGATACGGCGACCACCGAT
Idx4: TGTGTATCAGTACATGGAATGATACGGCGACCACCGAT
Idx5: ACACGCATGACACACTGAATGATACGGCGACCACCGAT
Idx6: GATCTCTACTATATGCGAATGATACGGCGACCACCGAT

C1-P1-T31: Bio-
AATGATACGGCGACCACCGATCGTTTTTTTTTTTTTTTTTTTTTTTTTTTTTT
T
C1-P1-RNA-TSO: Bio-AAUGAUACGGCGACCACCGAUNNNNNGGG (RNA)
